# Selective human inhibitors of ATR and ATM render *Leishmania major* promastigotes sensitive to oxidative damage

**DOI:** 10.1371/journal.pone.0205033

**Published:** 2018-09-28

**Authors:** Raíssa Bernardes da Silva, Carlos Renato Machado, Aldo Rogelis Aquiles Rodrigues, André Luiz Pedrosa

**Affiliations:** 1 Departamento de Bioquímica, Farmacologia e Fisiologia, Instituto de Ciências Biológicas e Naturais, Universidade Federal do Triângulo Mineiro, Uberaba, Minas Gerais, Brazil; 2 Departamento de Bioquímica e Imunologia, Instituto de Ciências Biológicas, Universidade Federal de Minas Gerais, Belo Horizonte, Minas Gerais, Brazil; Instituto Butantan, BRAZIL

## Abstract

All cellular processes, including those involved in normal cell metabolism to those responsible for cell proliferation or death, are finely controlled by cell signaling pathways, whose core proteins constitute the family of phosphatidylinositol 3-kinase-related kinases (PIKKs). Ataxia Telangiectasia Mutated (ATM) and Ataxia Telangiectasia and Rad3 related (ATR) are two important PIKK proteins that act in response to DNA damage, phosphorylating a large number of proteins to exert control over genomic integrity. The genus *Leishmania* belongs to a group of early divergent eukaryotes in evolution and has a highly plastic genome, probably owing to the existence of signaling pathways designed to maintain genomic integrity. The objective of this study was to evaluate the use of specific human inhibitors of ATR and ATM in *Leishmania major*. Bioinformatic analyses revealed the existence of the putative PIKK genes ATR and ATM, in addition to mTOR and DNA-PKcs in *Leishmania* spp. Moreover, it was possible to suggest that the inhibitors VE-821 and KU-55933 have binding affinity for the catalytic sites of putative *L*. *major* ATR and ATM, respectively. Promastigotes of *L*. *major* exposed to these inhibitors show slight growth impairment and minor changes in cell cycle and morphology. It is noteworthy that treatment of promastigotes with inhibitors VE-821 and KU-55933 enhanced the oxidative damage caused by hydrogen peroxide. These inhibitors could significantly reduce the number of surviving *L*. *major* cells following H_2_O_2_ exposure whilst also decreasing their evaluated IC_50_ to H_2_O_2_ to less than half of that observed for non-treated cells. These results suggest that the use of specific inhibitors of ATR and ATM in *Leishmania* interferes in the signaling pathways of this parasite, which can impair its tolerance to DNA damage and affect its genome integrity. ATR and ATM could constitute novel targets for drug development and/or repositioning for treatment of leishmaniases.

## Introduction

A large part of the life cycle of organisms involves dealing with compounds generated by an oxygenated environment. Reactive oxygen species (ROS) comprise many types of chemicals that can arise in different cell contexts, and include superoxide anions, hydroxyl radicals and hydrogen peroxide. From endogenous sources, ROS may be the result of normal intracellular metabolism, derived from processes in the cytosol or in organelles including the mitochondria and peroxisomes. ROS may result from exogenous sources including the action of physical agents such as ultraviolet light and ionizing radiation, chemical agents including environmental toxins and chemotherapeutics, and host inflammatory responses to infection [1, 2]. The oxidative stress caused by ROS may result in modifications to proteins, lipids or DNA. In nucleic acids, the oxidative damage includes base or sugar group adducts, single- and double-strand breaks, and crosslinks with other molecules. DNA adducts account for more than twenty known products in mammals, for example, modifications of all four bases and thymine-tyrosine crosslinks [3].

This wide variety of lesions that can affect genomic DNA integrity is managed by specialized repair pathways in cells, including mismatch repair (MMR), base excision repair (BER), nucleotide excision repair (NER), non-homologous end joining (NHEJ), and homologous recombination (HR) [4]. These lesions have also led to the existence of an intricate signaling network, which maintains cellular genomic integrity, namely through DNA damage response (DDR), a network coordinated by the PIKK proteins ATM and ATR. These proteins have different affinities for binding sites and recruitment forms. ATM binds preferably to double-strand break sites, recruited by the MRN complex (Mre11-Rad50-NBS1), whilst ATR binds to single-strand break sites, recruited by RPA heterotrimer (replication protein A). However, their functions are frequently linked, thus allowing them to control the DDR processes, which include transcription and activation of suitable proteins for DNA repair, cell cycle control, senescence, and apoptosis [5, 6].

The *Leishmania* genus belongs to the family Trypanosomatidae, a group of organisms that diverged early in eukaryote evolution. During their life cycle, these parasites are challenged by a wide variety of conditions when infecting different hosts and therefore, must have mechanisms that allow their adaptation, survival, and reproduction [7, 8]. With respect to their molecular and genomic organization, these protozoans have several unique features, which differentiate them from other eukaryotes. Perhaps the most striking feature is the organization of their genes in polycistronic transcription units. These units refer to groups of tens to hundreds of protein-coding genes arranged sequentially in the same DNA strand and, in this arrangement, transcribed into the same mRNA strand [9, 10]. To achieve control over the expression of their genes, these parasites depend upon mechanisms unlike those observed in other eukaryotes. These include the processing of transcripts by trans-splicing and polyadenylation [11] and more complex pathways, which involve direct modification of the genome. For example, cells selected to be resistant to cytotoxic compounds frequently amplify or delete a number of loci coding for drug targets or their transporters [12, 13]. The occurrence of this mechanism has been reported for several genes, including MRPA and ARM58 gene amplifications or aquaglyceroporin gene deletions in strains resistant to antimonial derivatives [14–16]. This appears to be a trait of the *Leishmania* genus, which reflects their high levels of genomic plasticity. The mechanisms of control and maintenance through which these protozoans are able to tolerate the major modifications to their genome remain to be determined.

Acknowledging the central role that these PIKK proteins have in metabolism coordination and the role of ATR and ATM in the maintenance of genomic integrity, the goal of this work was to evaluate the effect of human inhibitors of these two proteins in *Leishmania major* promastigotes. From computational data, we identified the putative ATR and ATM of *L*. *major*, and defined their possible three-dimensional structures and their ability to interact with specific inhibitor compounds. Moreover, the usage of these compounds allowed us to evaluate alterations in the growth patterns, morphology, and cell cycle of *L*. *major*. Finally, we also analyzed the behavior of *L*. *major* treated with ATR and ATM inhibitors when exposed to hydrogen peroxide, a ROS involved in oxidative responses against this parasite. Promastigote forms treated with human ATR and ATM inhibitors displayed higher susceptibility to oxidative damage, which may represent a novel strategy for the treatment of leishmaniases in the future.

## Materials and methods

### Parasite cultures

Promastigote forms of *Leishmania major* (*Lmj*) clone CC1 wild type were maintained in M199 medium supplemented with 10% fetal bovine serum, as previously described [17]. Cultures were kept in Bio-Oxygen Demand (BOD) incubators at 28°C, with medium replacement every seven days.

### Inhibitors

VE-821 (ATRi) provided by J. R. Pollard (Vertex Pharmaceuticals, UK) was used for *L*. *major* growth curves in the presence of inhibitors and for cytometry and morphological analyses. Subsequently, the drug was commercially purchased from Sigma-Aldrich (MO, USA), as well as KU-55933 (ATMi) and caffeine, which were used for survival trials following exposure to H_2_O_2_.

ATRi and ATMi were previously diluted in DMSO, the final concentration of which [maximum of 0.4% (v/v)] did not interfere with any of the described experiments. Caffeine was previously diluted in M199 medium, since the large volume required for dilutions could significantly alter the concentrations of the medium components. Stock solutions were kept at -20°C and protected from light to prevent degradation.

### Growth curves of *L*. *major* treated with inhibitors

*L*. *major* promastigote forms were collected during log-phase and inoculated at a concentration of 2 × 10^5^ cells/mL in 24-well plates containing standard M199 medium and caffeine, ATRi, ATMi, or a combination of these drugs in various concentrations. The parasite growth curves were determined with daily counts, recorded at the same time using a hemocytometer, until the treated cells reached the stationary phase, characterized by three days of similar counts. All cultures were performed in triplicate and data is expressed as the mean ± standard error of the triplicates.

### Survival assay after exposure to H_2_O_2_ and determination of IC_50_

To determine the dose-response effect of human ATR and ATM inhibitors on *L*. *major* exposed to H_2_O_2_, log-phase promastigote forms were collected and centrifuged at 2,000 *g* for 10 minutes at 4°C for complete removal of the culture medium. Then, they were resuspended to a concentration of 5 × 10^6^ cells/mL in M199 medium containing different concentrations of inhibitors, obtained by serial dilution. The samples were incubated for 1 hour at 28°C prior to the induction of oxidative damage by the addition of 500 μM H_2_O_2_ (Sigma-Aldrich, MO, USA) [18, 19].

To determine the inhibitory concentration of H_2_O_2_ for 50% of cell growth (IC_50_), log-phase promastigotes were collected and centrifuged at 2,000 *g* for 10 minutes at 4°C for complete removal of the culture medium. Then, they were resuspended to a concentration of 5 × 10^6^ cells/mL in M199 medium containing 10 μM ATRi, 10 μM ATMi, or 5 mM caffeine. The samples were incubated for 1 hour at 28°C prior to the addition of H_2_O_2_ at concentrations of 0, 500, 1000, 1500, 2000 or 2500 μM.

For both procedures, the cells were exposed to H_2_O_2_ for 20 minutes, without agitation, and protected from light to prevent H_2_O_2_ decomposition. After this time, they were centrifuged at 2,000 g for 10 minutes at 4°C and the supernatant was completely washed out and replaced with the same volume of standard M199. The cells were distributed in 24-well plates in triplicate, protected from light, and counted after 72 hours with a hemocytometer. One hundred percent of growth was recorded for cell lines not treated with inhibitors nor exposed to H_2_O_2_; the percentage growth for the other treatment groups was relative to this control. Data are presented as mean ± standard error of the relative growths or IC_50_s obtained from three independent experiments.

### Cell cycle analysis by flow cytometry

*L*. *major* promastigote forms were collected during log-phase and inoculated at a concentration of 2 × 10^5^ cells/mL in 75-cm^2^ bottles containing standard M199 medium and either caffeine (1.25 mM, 5 mM, and 20 mM), ATRi (2.5 μM, 10 μM, and 40 μM), ATMi (2.5 μM, 10 μM, and 40 μM), or a combination of these drugs in various concentrations. Cells (0.5 to 2 × 10^7^) were collected from the cultures shortly after the inoculum and after 24 h, 48 h, and 72 h of incubation with the inhibitors. Samples were washed twice with 1× PBS and fixed with 70% (v/v) methanol in 1× PBS overnight at 4°C. Fixed cells were washed with 1× PBS and labeled with 50 μg/mL propidium iodide, 100 μg/mL RNase A in 1× PBS at 37°C for 45 minutes [20]. Flow cytometry data were collected using a FACScan BD flow cytometer and data for 10,000 events were analyzed using FlowJo software version 10.0.7.

### Fluorescence microscopy

To determine the effect of human ATR and ATM inhibitors on cell division events of *L*. *major*, *L*. *major* promastigote forms were collected during log-phase and inoculated at a concentration of 5 × 10^6^ cells/mL in 24-well plates containing standard M199 medium and caffeine, ATRi, ATMi, or a combination of these drugs in various concentrations. After 24 hours of incubation at 28°C, protected from light, a volume of 200 μL was collected from each well. Cells were washed with 1× PBS and resuspended in 100 μL 1× PBS. Then, 50 μL of each sample was dispensed individual into glass slides and left for 10 minutes for cell adhesion. The supernatant was removed and replaced with 4% paraformaldehyde diluted in 1× PBS for fixation for 15 minutes. The supernatant was then removed and the wells were washed three times with 1× PBS prior to the addition of Hoescht 33342 solution (Thermo Fisher Scientific, MA, USA), diluted 1:1000 in 1× PBS. Following incubation for 1 minute, the dye was removed and the wells were washed three times with 1× PBS. The glass slides were finally mounted with VectaShield Antifade Mounting Medium (Vector Laboratories, CA, USA). Images were acquired with the Zeiss Apotome 2 and Zeiss LSM-710 fluorescence microscopes. From each sample, 500 cells were counted and each cell was classified according to its phenotype: 1N/1K, 1N/2K, 2N/2K, 1N/0K and 0N/1K. Owing to the limitations of the technique, 1N/0K and 0N/1K cell types were collectively classified as “aberrant forms”, and this nomenclature was used for the following statistical analyses.

### Search for PIKK protein sequences in *Leishmania major*

The human ATR protein sequence (GenBank accession number NP_001175.2) was analyzed using the NCBI CD-Search conserved domain search tool [21]. The catalytic domain of PIKK was defined as ranging from amino acid 2293 to 2567, (a total of 275 amino acids), and its sequence was used for a non-redundant protein database search in the *Leishmania major* MHOM/IL/81/Friedlin genome, using the PSI-BLAST algorithm [22]. Then, the sequences recovered from *L*. *major* were used to do a reciprocal search in the non-redundant protein database in the *Homo sapiens* genome, using BLASTP, and the top hit for each one was selected.

### Construction of phylogenetic tree of PIKK proteins

With each of the PIKK protein sequences retrieved from *L*. *major* genome, we searched for orthologous proteins in six other *Leishmania* species, (*Leishmania infantum*, *Leishmania donovani*, *Leishmania mexicana*, *Leishmania guyanensis*, *Leishmania panamensis* and *Leishmania braziliensis*), and trypanosomatids (genus *Leptomonas* and *Trypanosoma*), as well as model organisms (*Homo sapiens*, *Mus musculus*, *Saccharomyces cerevisiae*, *Caenorhabditis elegans*, *Arabdopsis thaliana*, *Drosophila melanogaster*). The search was performed using the PSI-BLAST algorithm and all rescued orthologous sequences that were used in the later steps can be found in S1 Table. The selected sequences were analyzed with CD-Search to delimit the catalytic domain of each PIKK (S1 Table). To construct the phylogenetic tree, the delimited sequences of the domains were aligned using the MUSCLE algorithm. The alignment was then trimmed and spurious sequences were removed using the automatic trimAI algorithm [23, 24]. The maximum likelihood phylogenetic tree was obtained using MetaPIGA v.3.1 and the bootstrap consensus tree was inferred from 1000 replicates [25].

### Analysis and construction of maps of the putative *Lmj*ATR and *Lmj*ATM protein domains

To construct the putative *Lmj*ATR and *Lmj*ATM domain maps, the sequences of these proteins were analyzed using Hierarchical Neural Networks (HNN– https://www.expasy.org/tools) to predict their secondary structures [26]. The same procedure was carried out for proteins of selected species: *L*. *infantum*, *L*. *braziliensis*, *T*. *brucei*, *T*. *cruzi*, *H*. *sapiens* and *S*. *cerevisiae*. To verify the position of the predicted domains in relation to the general secondary structure of the proteins, the maps containing the predicted regions for α-helix, β-strand and loops were manually aligned with the domain predictions from CD-Search.

### Analysis of the tertiary structures of the catalytic domains of putative *Lmj*ATR and *Lmj*ATM and molecular docking

The tertiary structure models of the putative *Lmj*ATR and *Lmj*ATM proteins were constructed by combined homology and *ab initio* strategies using the ROBETTA server (http://robetta.bakerlab.org/) and the tertiary structure of human mTOR as a template under RCSB Protein Data Bank (PDB) accession code 5FLC [27, 28]. Five models were constructed for each protein, which were then analyzed for selection of the best model and validated by the parameters analyzed by the MolProbity tool (http://molprobity.biochem.duke.edu/) [29]. The chosen models were used in subsequent analyses.

The chosen structures from the catalytic domains of the putative *Lmj*ATR and *Lmj*ATM proteins were aligned with the experimentally determined three-dimensional structures of *Hs*ATR (PDB ID: 5YZ0 [30]) and *Hs*ATM (PDB ID: 5NP0 [31]), respectively, using the Protein Structure Comparison Tool v. 4.2.0 and the combinatorial extension algorithm (jCE) [32, 33]. The structural alignments were analyzed with Jpred to estimate the amino acid and secondary structure consensus, and the data were visualized in Jalview v.2.10.3b1 [34, 35].

The molecules of ATRi and ATMi were docked into the respective targets, the putative *Lmj*ATR and *Lmj*ATM proteins, with Autodock v. 4.2.6, and the conformational states with the highest free energy of binding released were selected [36]. The interactions of the proteins with the compounds were visualized using Chimera v. 1.11.2 [http://www.rbvi.ucsf.edu/chimera/] and Autodock Tools v. 1.5.6 [http://autodock.scripps.edu/resources/adt] [36, 37].

### Statistical analyses

All statistical analyses were performed using GraphPad Prism v. 5.03 and all tests results were considered significant when the p-value was less than 0.05. Growth curves with the inhibitors were analyzed using nonlinear regression and the extra squares sum F test. Flow cytometry data (percentage of cells at each stage of the cell cycle: <G1, G1, S, G2 and >G2) and morphology data (percentage of cells with each phenotype evaluated) were analyzed using the chi-squared test. The IC_50_s for H_2_O_2_ were calculated using nonlinear regression and the curves used for the calculation can be seen in S3 Fig. With the values obtained, analysis of variance (ANOVA) and Dunnett post-hoc tests were applied, using values for non-treated cells as the control. To determine the dose-response effect of ATRi and ATMi post-exposure to H_2_O_2_, data collected from three independent experiments were normalized relative to the respective control (i.e. non-treated and not exposed to H_2_O_2_). For the exposed or unexposed H_2_O_2_ groups, the Shapiro-Wilk test was applied for normality evaluation, as well as ANOVA followed by the Dunnett post-hoc test.

## Results

### Putative PIKK family proteins present in *Leishmania* genome

Protein kinases belonging to the PIKK family display sequence similarity with the phosphatidylinositol 3-kinases and are evolutionarily conserved, being found in many classes of organisms [38]. We used the catalytic domain sequence of human ATR, a segment of 275 amino acids in the C-terminal region of this protein, to search the sequences of putative members of the PIKK family in *L*. *major*. From the results, we selected five *L*. *major* sequences (*Lmj*F.32.1460, *Lmj*F.02.0120, *Lmj*F.36.6320, *Lmj*F.34.4530, *Lmj*F.36.2940), to search within the human protein databank. From reciprocal BLAST analyses, these *L*. *major* sequences retrieved human orthologs identified as ATR, ATM, mTOR and DNA-PKcs (Table 1).

**Table 1:** Identification of putative ATR (Ataxia telangiectasia and Rad3 related), ATM (Ataxia telangiectasia mutated), mTOR (mechanistic Target of Rapamycin) and DNA-PKcs (DNA-dependent protein kinase, catalytic subunit) *Leishmania major* protein sequences.

*Leishmania major* rescued protein sequences^a^					Reciprocal BLASTP in *Homo sapiens* reference protein databank^b^		
Description	Accession^e^	Protein size^c^	Domain size^c^ (range^d^)	E-value	Description	Accession^f^	E-value
Putative phosphatidylinositol 3-related kinase	*Lmj*F.32.1460	3207	273 (2863–3135)	1e-77	Serine/threonine-protein kinase ATR	NP_001175.2	1e-92
Putative phosphatidylinositol k**i**nase related protein	*Lmj*F.02.0120	4905	281 (4537–4817)	2e-50	Serine/threonine-protein kinase ATM	NP_000042.3	4e-89
Putative target of rapamycin kinase 1	*Lmj*F.36.6320	2613	277 (2131–2407)	4e-49	Serine/threonine-protein kinase mTOR	NP_004949.1	4e-148
Putative target of rapamycin kinase 2	*Lmj*F.34.4530	2438	285 (2033–2317)	4e-42	Serine-threonine-protein kinase mTOR	NP_004949.1	5e-151
Conserved hypothetical protein	*Lmj*F.36.2940	4183	304 (3799–4102)	1e-22	DNA-dependent protein kinase catalytic subunit	NP_008835.5	1e-25

(a) *L*. *major* sequences were rescued in a PSI-BLAST search using the conserved PIKK (phosphatidylinositol 3-kinase related kinase) domain of human ATR as a query (GenBank accession number NP_001175.2, residue range 2293–2567).

(b) *L*. *major* sequences with significant E-values with the human-ATR-PIKK domain were used as queries in a BLASTP search in the non-redundant protein sequences databank, restricted to *Homo sapiens* species.

(c) Protein size, in residues.

(d) Range of residue positions of the rescued proteins PIKK domain, as predicted by CD-SEARCH.

(e) TriTryp databank (http://tritrypdb.org/tritrypdb/) accession number.

(f) GenBank (https://www.ncbi.nlm.nih.gov/genbank/) accession number.

**Fig 1:**
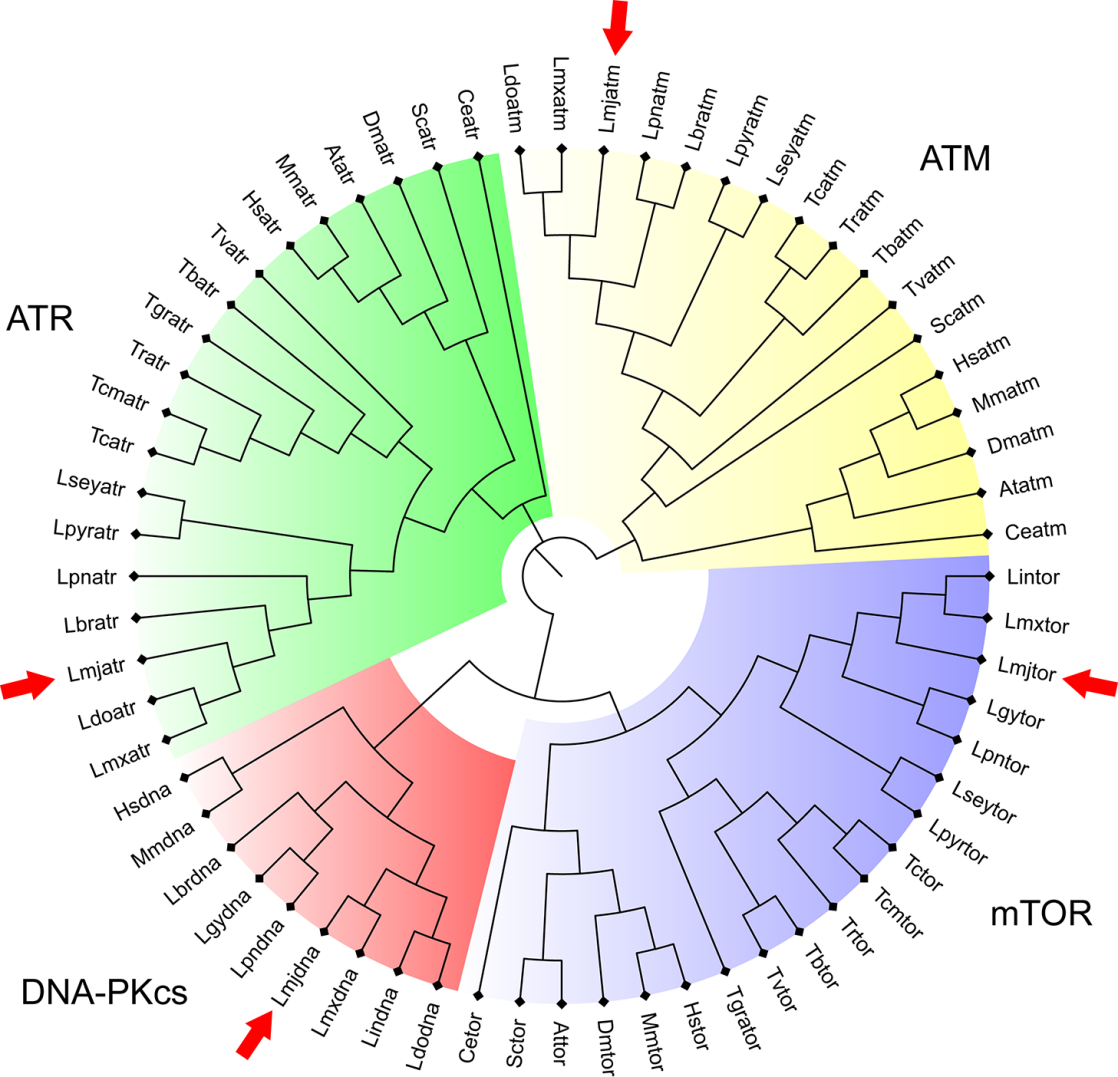
Phylogenetic analysis of *Leishmania major* PIKK putative proteins. Radial cladogram represents the alignment of PIKK domains of putative ATR (green), ATM (yellow), mTOR (blue), and DNA-PKcs (red) of trypanosomatids and other eukaryotes. *Leishmania major* sequences are highlighted by the red arrows. Lmj: *Leishmania major*; Lin: *Leishmania infantum*; Ldo: *Leishmania donovani*; Lmx: *Leishmania mexicana*; Lgy: *Leishmania guyanensis*; Lpn: *Leishmania panamensis*; Lbr: *Leishmania braziliensis*; Lse: *Leptomonas seymori*; Lpy: *Leptomonas pyrrhocoris*; Tgr: *Trypanosoma grayi*; Tbr: *Trypanosoma brucei*; Tcr: *Trypanosoma cruzi*; Tcm: *Trypanosoma cruzi marinkellei*; Tvi: *Trypanosoma vivax*; Tra: *Trypanosoma rangeli*; Hsa: *Homo sapiens*; Mmu: *Mus musculus*; Sce: *Saccharomyces cerevisiae*; Cel: *Caenorhabditis elegans*; Ath: *Arabidopsis thaliana*; Dme: *Drosophila melanogaster*. The displayed tree represents the bootstrap consensus of 1000 non-parametric replicates, visualized with FigTree v1.4.3. The complete list of sequences used for this analysis is available in S1 Table. Orthologs for some species were not found during the BLAST analysis. As some domain sequences between species were identical, only one sequence of each ortholog was kept for further phylogenetic analysis. In these cases the sequence with the lowest number of ambiguities was kept. The identical sequences were: *Leishmania guyanensis* and *Leishmania panamensis* ATR domains; *Leishmania infantum* and *Leishmania donovani* ATR domains; *L*. *infantum* and *L*. *donovani* mTOR domains; *Leishmania braziliensis* and *L*. *guyanensis* mTOR domains; *L*. *infantum* and *L*. *donovani* ATM domains; and *L*. *guyanensis* and *L*. *panamensis* ATM domains.

Using these *L*. *major* PIKK protein sequences, we could also identify their homologs in other species of *Leishmania* genus and related trypanosomatids, including *Leptomonas* spp., *Trypanosoma cruzi*, and *Trypanosoma brucei* (Fig 1). Owing to the high degree of conservation, we performed a phylogeny analysis using the catalytic domain sequences of the encountered PIKK proteins (S1 and S2 Tables). Within each PIKK protein group, trypanosomatid proteins related more closely to each other in comparison to that of their orthologs in higher eukaryotes. Also of note is that amongst the analyzed PIKK proteins ATR, ATM, and mTOR, orthologs can be found amongst many eukaryote species, but not for DNA-PKcs orthologs. We have not identified DNA-PKcs orthologs within other trypanosomatid genera besides *Leishmania*. This could indicate that this PIKK protein, related to the NHEJ repair pathway, could represent functions in the *Leishmania* genus that are not found in other trypanosomatids.

### Identification of the structural domains of putative ATR and ATM of *L*. *major*

**Fig 2:**
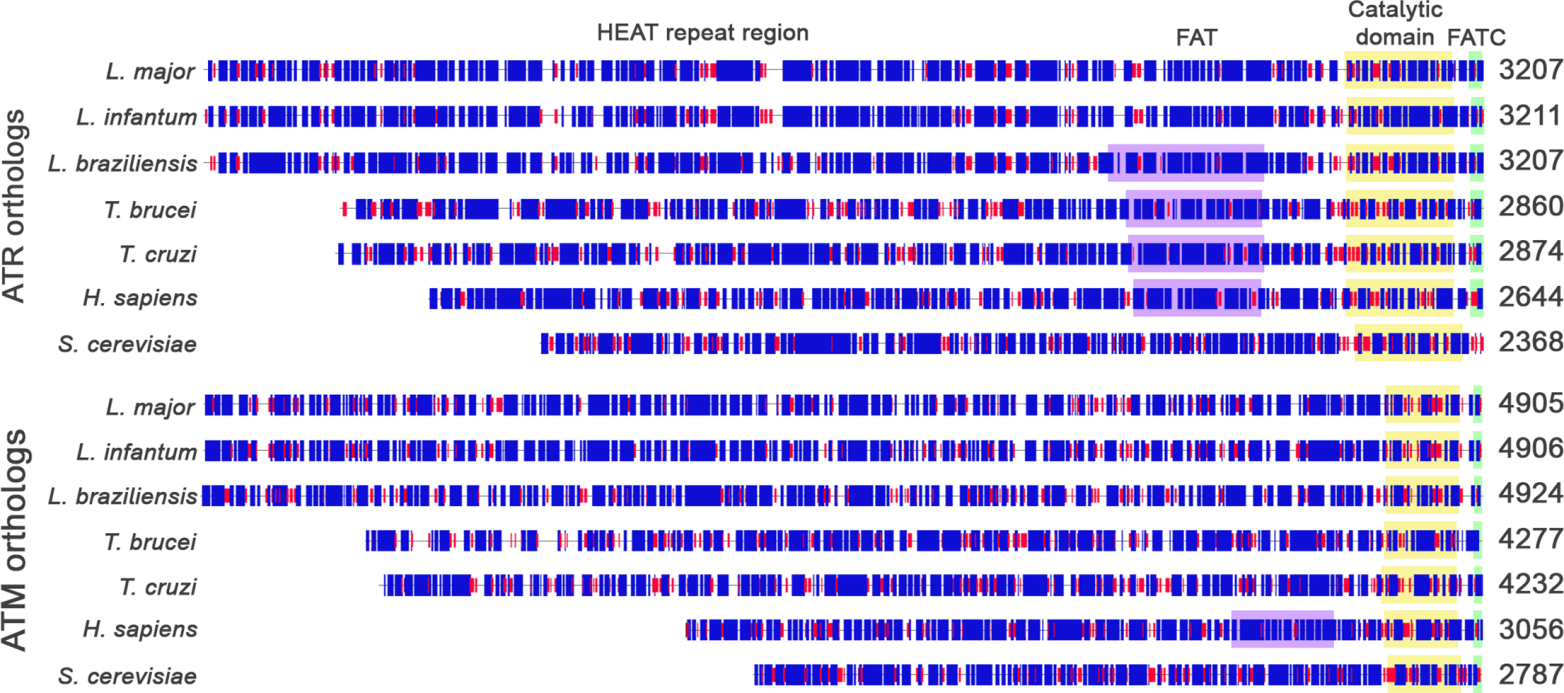
Map of main domains of ATR and ATM identified orthologs. Complete sequences of the orthologs were analyzed with Hierarchical Neural Networks (HNN– https://www.expasy.org/tools) and the last residue of each protein was used to align the resulting maps. The main conserved domains of PIKK proteins were predicted by CD-Search and are indicated as FAT (FRAP, ATM, TTRAP—purple box); catalytic domain (yellow box) and FATC (FRAP, ATM, TTRAP C-terminal—green box). The HEAT repeat region, is believed to be present at the N-terminal of ATR and ATM. Numbers display protein sizes and predicted secondary structures are indicated by blue boxes for alpha-helices and red boxes for beta-strands.

The predicted ATR and ATM proteins of *L*. *major* have 3207 and 4905 amino acids, and predicted molecular weights of approximately 351 kDa and 524 kDa, respectively (Fig 2). As shown in Fig 2, we aligned the homologs of ATR and ATM by the last amino acid residues, which highlights the similarities of the C-terminal region. At this protein site, the catalytic, flanking FAT and FATC domains have similar sizes and positions according to domain predictors. However, differences are evident in the N-terminal portion of these proteins. This region, predominantly comprised of alpha-helices (approximately 57% for *Lmj*ATR and 50% for *Lmj*ATM), covers the HEAT (huntingtin, elongation factor 3, A subunit of protein phosphatase 2A and TOR1) repeats [39]. For ATR, *Leishmania* spp. has an average of 341 more amino acid residues than the *Trypanosoma* spp. *Trypanosoma* spp. also has 223 and 499 residues more than *Homo sapiens* and *Saccharomyces cerevisiae*, respectively. For ATM, the differences are greater, *Leishmania spp*. exhibits 658 more amino acids than *Trypanosoma* spp., which has 1198 and 1467 more amino acids than that in humans and yeast, respectively. The large differences in length and sequence of the N-terminus (S2 Table) may indicate differences in the number of HEAT repeats present. These differences may have implications for how these proteins interact with their targets and consequently, how they will react to various types of DNA damage.

**Fig 3:**
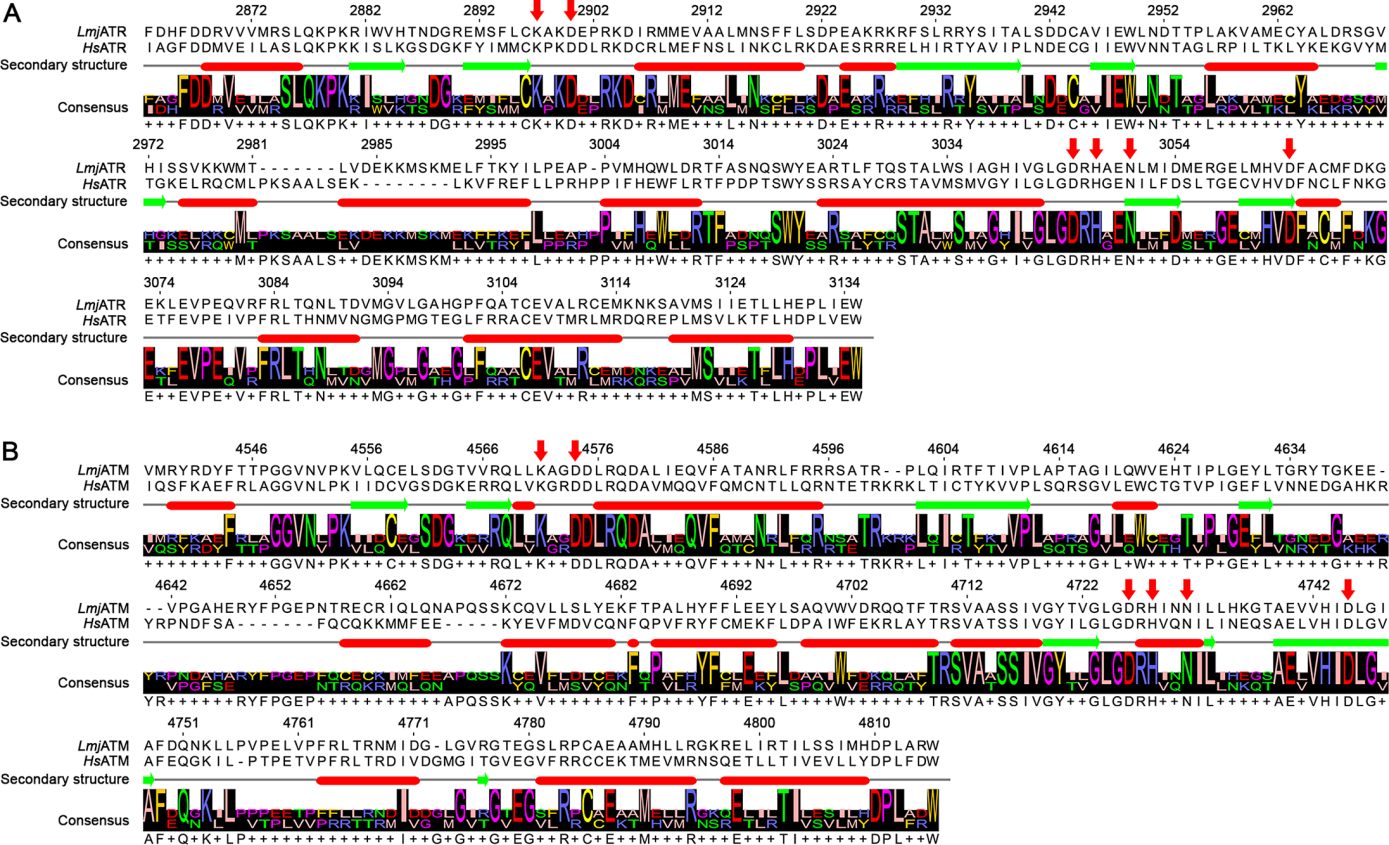
Structure alignment of *L*. *major* and human ATR and ATM. Alignment of *L*. *major* and human ATR (A) and ATM (B) protein sequences according to their secondary structure. Secondary structures are indicated by red ovals for alpha-helices and green arrows for beta-sheets; gaps in the alignment are indicated as dashes (-), more than one possible amino acid in the consensus is indicated by a plus sign (+). Red arrows above the sequences indicate important residues for the catalytic activity of the proteins; the numbers above the sequence indicate the position of that amino acid in the corresponding *L*. *major* protein. Amino acids residues are presented as International Union of Pure and Applied Chemistry (IUPAC) one-letter notation code.

As stated previously, the C-terminal region of these proteins appears to hold the majority of their sequence and structural conservation. To further analyze this feature and infer the compatibility of human inhibitory compounds in *Leishmania*, we generated three-dimensional models of the catalytic domain of putative *Lmj*ATR and *Lmj*ATM. The generated models were aligned structurally with their respective human homologs, displaying similar folding with root-mean-squared deviations (RMSD) of 2.09 Å for *Lmj*ATR and 2.67 Å for *Lmj*ATM. In the putative *Lmj*ATR protein, the main residues predicted to be related to catalytic activity are conserved in their human counterpart: K2897 and D2900 for ATP association; N3050 and D3064 stabilize Mg^2+^ for catalysis; D3045 for activation of the hydroxyl group in the substrate for nucleophilic attack; and H3047 for electrostatic stabilization of the transition state (Fig 3A) [30]. The putative *Lmj*ATM also shares with *Hs*ATM the residues involved in kinase activity: K4571, D4574, H4728, D4726, N4731 and D4745, predicted to bind to ATP or Mg^2+^ ion (Fig 3B) [40].

### Molecular docking of ATRi and ATMi in the putative *L*. *major* ATR and ATM protein kinases

**Fig 4:**
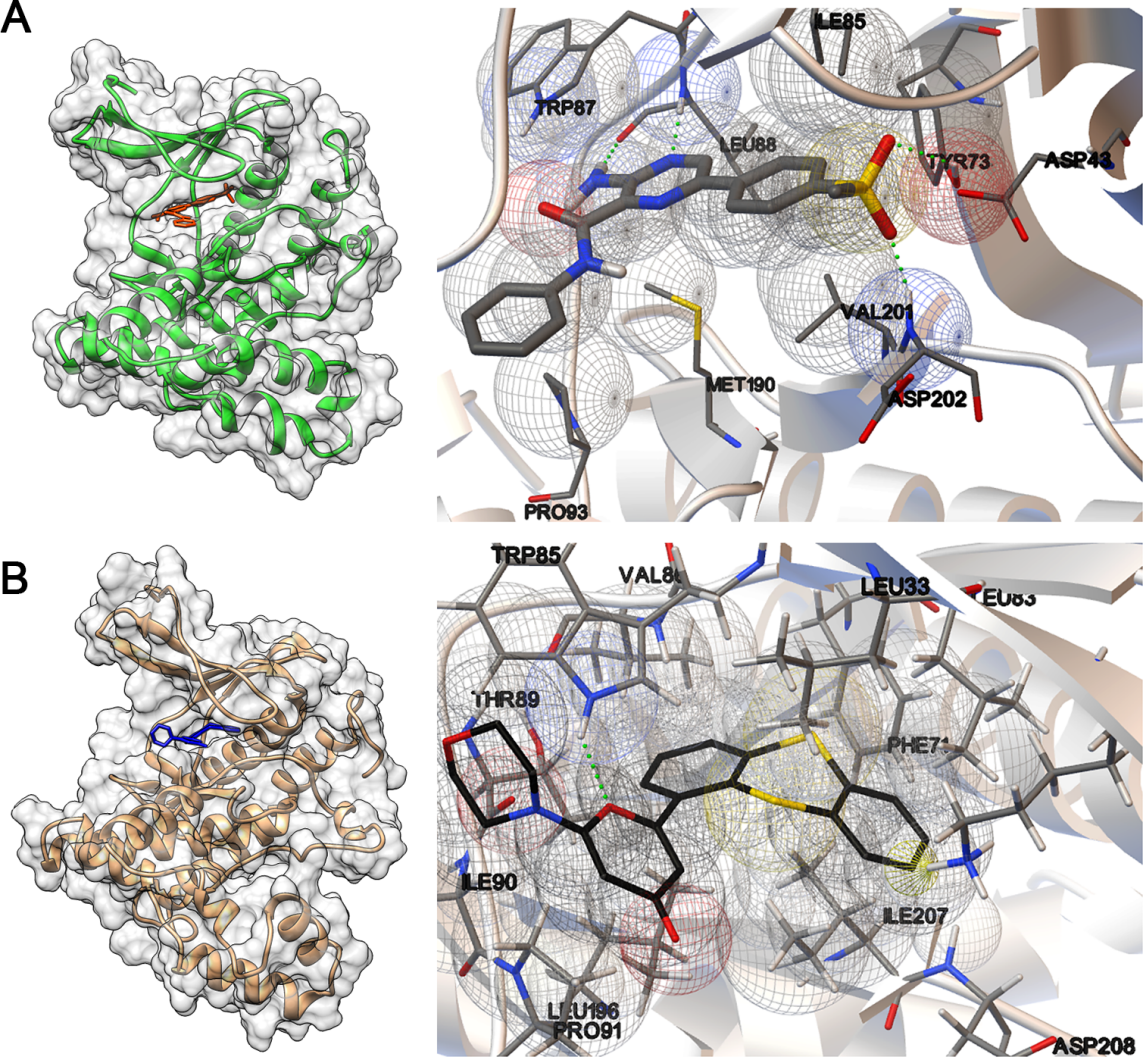
View of interaction site of putative *Lmj*ATR and *Lmj*ATM with the human inhibitors. Left panels: Molecular modeling of the C-terminal region of *Lmj*ATR (A) and *Lmj*ATM (B). The sequences used for the modeling correspond to the predicted PIKK domains and range from amino acids 2863 to 3135 and 4537 to 4817 in *Lmj*ATR and *Lmj*ATM, respectively. The catalytic domains of *Lmj*ATR and *Lmj*ATM are displayed with the corresponding human inhibitors, VE-821 (ATRi—red) and KU-55933 (ATMi—blue). Right panels: View of respective docking sites. The compound structures and residue side chains predicted to participate in the interaction are displayed as sticks. ATRi is shown as thicker sticks and ATMi is shown as black sticks. Green dotted lines indicate hydrogen bonds, wireframe yellow cone indicates pi-cation interaction and wireframe spheres indicate Van der Waals interactions. Protein tertiary structure prediction was performed with ROBETTA and docking was performed with Autodock v. 4.2.6. The interactions of proteins and compounds were visualized with Chimera v. 1.11.2 (Left side) [http://www.rbvi.ucsf.edu/chimera/] and Autodock Tools v. 1.5.6 (right side) [http://autodock.scripps.edu/resources/adt].

Several ATR and ATM inhibitory compounds have been explored as therapeutic alternatives for cancer treatment, either as chemo- or radiosensitizers, or individually through synthetic lethality [41]. We chose to test human ATR and ATM-specific inhibitors, VE-821 (ATRi) and KU-55933 (ATMi), to verify their effect on *L*. *major* cells. We used molecular docking to show that these inhibitors have the putative ability to associate with their specific targets. ATRi and ATMi could occupy the space corresponding to the catalytic sites of putative *Lmj*ATR and *Lmj*ATM proteins (Fig 4A and 4B, left panels), in a competitive manner with the ATP molecule [42, 43]. Owing to the specific active site conformations and atom clashes, it was not possible to dock ATRi and ATMi into the opposite target. ATRi could be associated with *Lmj*ATR, releasing an estimated free energy of binding of -8.02 kcal/mol with an estimated inhibition constant Ki of 1.33 μM at 25°C. From interaction predictors, this molecule appears to form four hydrogen bonds with residues W2949, L2950, Y2935, D3064 (W87, L88, Y73 and D202 in the model), and Van der Waals interactions with I2947, P2955, M3052, V3063 (I85, P93, M190 and V201 in the model, Fig 4A, right panel). When associated with the putative *Lmj*ATM, ATMi releases an estimated free energy of binding of -8.85 kcal/mol, with an estimated Ki of 323.03 nM at 25°C. ATMi appears to form a hydrogen bond with residue W4621 (W85 in the model), a pi-cation interaction with I4743 (I207 in the model), and Van der Waals interactions with T4625, I4926, V4622, L4732, P4627, L4569, L4619, F4607 and D4744 (T89, I90, V86, L196, P91, L33, L83, F71 and D208 in the model, Fig 4B, right panel).

### Effects of human ATR and ATM inhibitors on the proliferation and cell cycle of *L*. *major*

**Fig 5:**
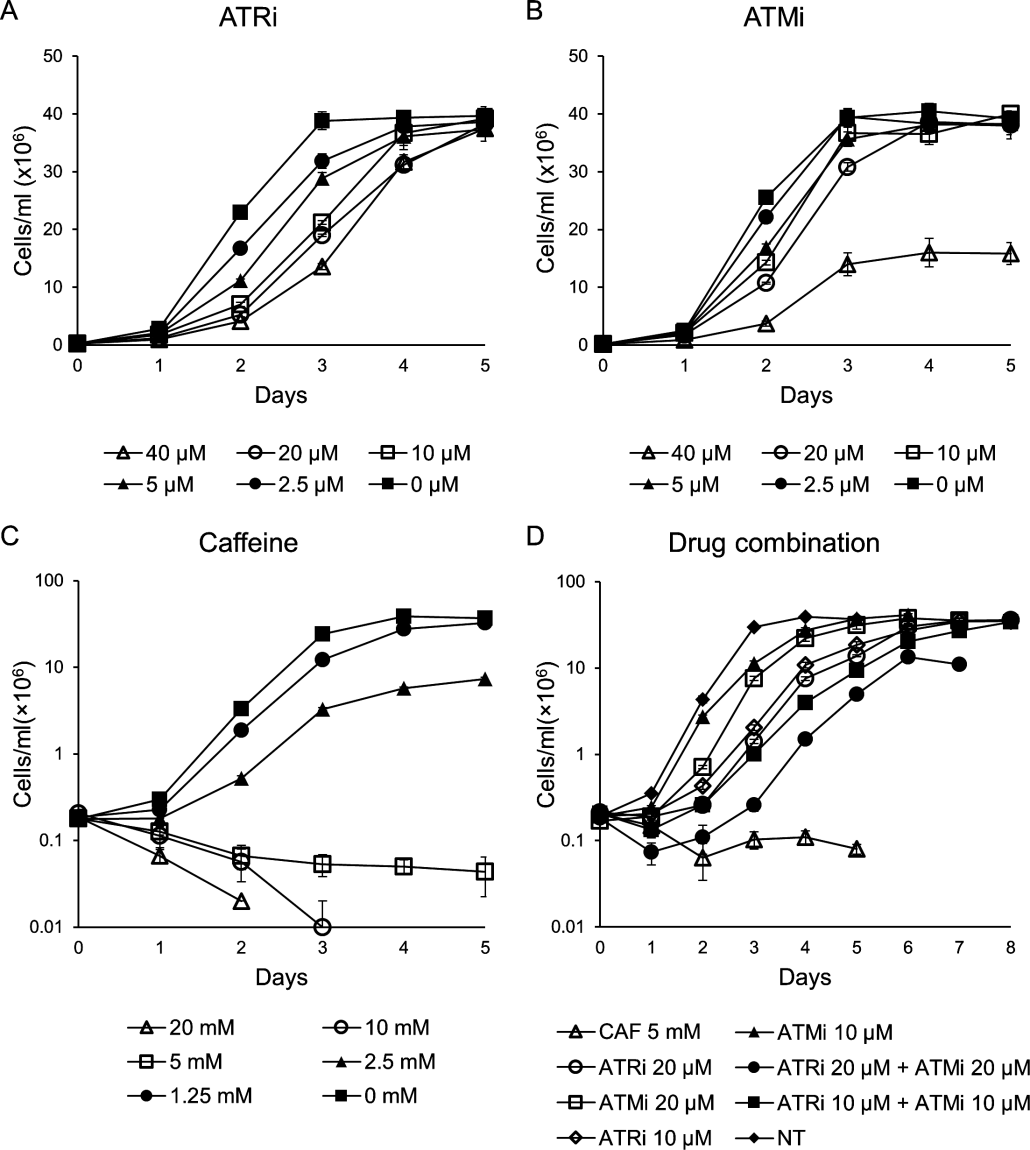
Growth curves of *L*. *major* promastigote forms exposed to ATRi, ATMi, caffeine, and combination of ATRi and ATMi. Log phase *L*. *major* promastigotes (2.0 × 10^5^ cells/mL) were inoculated in culture media containing increasing concentrations of ATRi (0; 2.5; 5; 10; 20; 40μM–A), ATMi (0; 2.5; 5; 10; 20; 40μM–B), caffeine (0; 1.25; 2.5; 5; 10; 20mM–C), or combinations of ATRi and ATMi (10μM ATRi + 10μM ATMi; 20μM ATRi + 20μM ATMi–D). Cells were counted every day during five- to eight-day periods, until they reached a stationary phase. Data represent mean ± SEM of triplicate samples and are representative of at least two independent experiments.

We verified the behavior of promastigotes in axenic culture of *L*. *major* when cultured in the presence of ATRi and ATMi (Fig 5). For comparison purposes, we used caffeine, a methylxanthine recognized as a non-selective inhibitor of ATR and ATM [44, 45]. We observed a dose-dependent reduction in the growth rate of promastigotes over five days, for both ATRi (Fig 5A) and ATMi (Fig 5B). When maintained in culture media containing ATRi or ATMi, the cells had doubling times up to 1.46 times longer than that observed for non-treated cells (NT cells doubling time: ≈ 8 hours; ATRi 40 μM doubling time: 11.3 hours; ATMi 40 μM doubling time: 11.7 hours), suggesting a role for these kinases in *L*. *major* replication. Growth arrest occurred for cells maintained in medium containing 5 mM caffeine. Cell death was observed in up to three days for caffeine concentrations above 5 mM (Fig 5C).

Following the logic that caffeine would simultaneously inhibit ATR and ATM, given its non-specific activity, we attempted to reproduce its effect on *L*. *major* by simultaneous addition of ATRi and ATMi (Fig 5D). We observed that the two drug combinations used (10 μM ATRi + 10 μM ATMi and 20 μM ATRi + 20 μM ATMi), increased the inhibitory effect on cell growth, causing growth arrest (10 μM ATRi + 10 μM ATMi), or death (20 μM ATRi + 20 μM ATMi), during the first 48 hours. However, after this period, the cells continued to grow, reaching a plateau similar to that in non-treated cells or with a slight reduction of maximum growth (when using the combined treatment of 20 μM ATRi + 20 μM ATMi). Therefore, the combination of the two ATR-specific and ATM-specific drugs at the concentrations used was able to elicit the caffeine effect at the 5 mM dose (i.e., complete growth arrest during the entire period), within the first 48 hours. However, after 48 hours, the drug combinations allowed cells to resume growth, unlike caffeine-treated cells, which demonstrates the potential of caffeine to inhibit kinases other than ATR and ATM that are also important for *L*. *major* replication.

**Fig 6:**
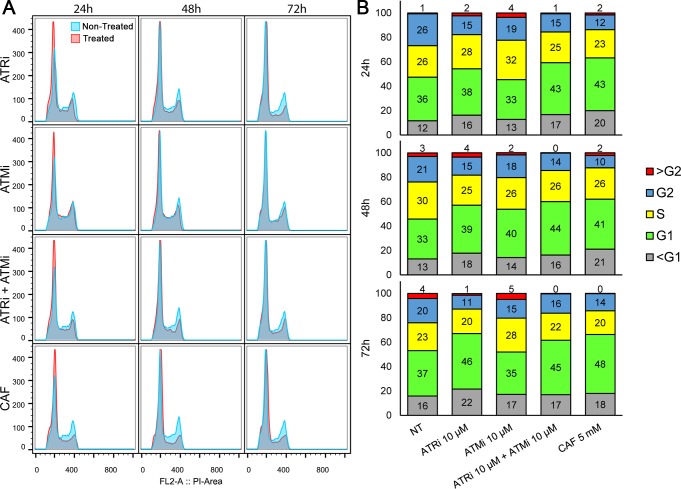
Cell cycle analysis of *L*. *major* promastigote forms exposed to ATRi, ATMi, and caffeine. (A) Log phase *L*. *major* cells were maintained in culture media containing 5 mM caffeine (CAF), 10 μM ATRi (VE-821), 10 μM ATMi (KU-55933), or a combination of 10 μM ATRi and 10 μM ATMi, and were compared to a non-treated control (NT). Samples (0.5 to 2 × 10^7^ cells) were collected after 24 h, 48 h, and 72 h of incubation with the inhibitors. Treated cells are displayed as red curves whilst non-treated cells are displayed as blue curves. (B) Data for 10,000 events were analyzed and stacked columns charts display percentages of cells in each cell cycle stage for non-treated cells (NT) and cells treated with 10 μM ATRi, 10 μM ATMi, combination of 10 μM ATRi and 10 μM ATMi, or 5 mM CAF, after 24, 48, or 72 hours of treatment. The evaluated cell cycle stages are G1 (green), S (yellow), G2 (blue), sub-G1 (grey) or above G2 (red). Numbers inside boxes indicate the respective percentages of each stage. Flow cytometry data were analyzed with FlowJo v. 10.0.7 and are representative of at least two independent experiments.

We also evaluated the effect of these inhibitors on the cell cycle of *L*. *major*, considering their effects on cell proliferation. Starting with cells under the same conditions (S1 Fig, 0 h), we observed a discrete increase in the proportion of cells retained in G1/S and a decrease in the number of cells in G2, following 24 hours of treatment with the inhibitors in comparison with that for non-treated cells. These effects intensified up to 48 hours post-treatment and appeared to be dose-dependent, with the highest proportions of cells retained in G1/S achieved by the highest concentrations of inhibitors used (Fig 6A and 6B, S1 Fig, S3 Table). Additionally, the use of inhibitor combinations (ATRi + ATMi), increases the differences in the cell cycle phase proportions, making their effects comparable to those obtained with the use of 5 mM caffeine (Fig 6B, S3 Table). The greatest discrepancies were observed with the use of 20 mM caffeine. In this case, the effect was the opposite, with a large increase in the number of cells retained at G2 (after 24 hours, increase from 26.1% to 52.3%), and a decrease in the number of G1 cells (from 35.6% to 23.2%) and S cells (from 25.9% to 16.0%).

**Fig 7:**
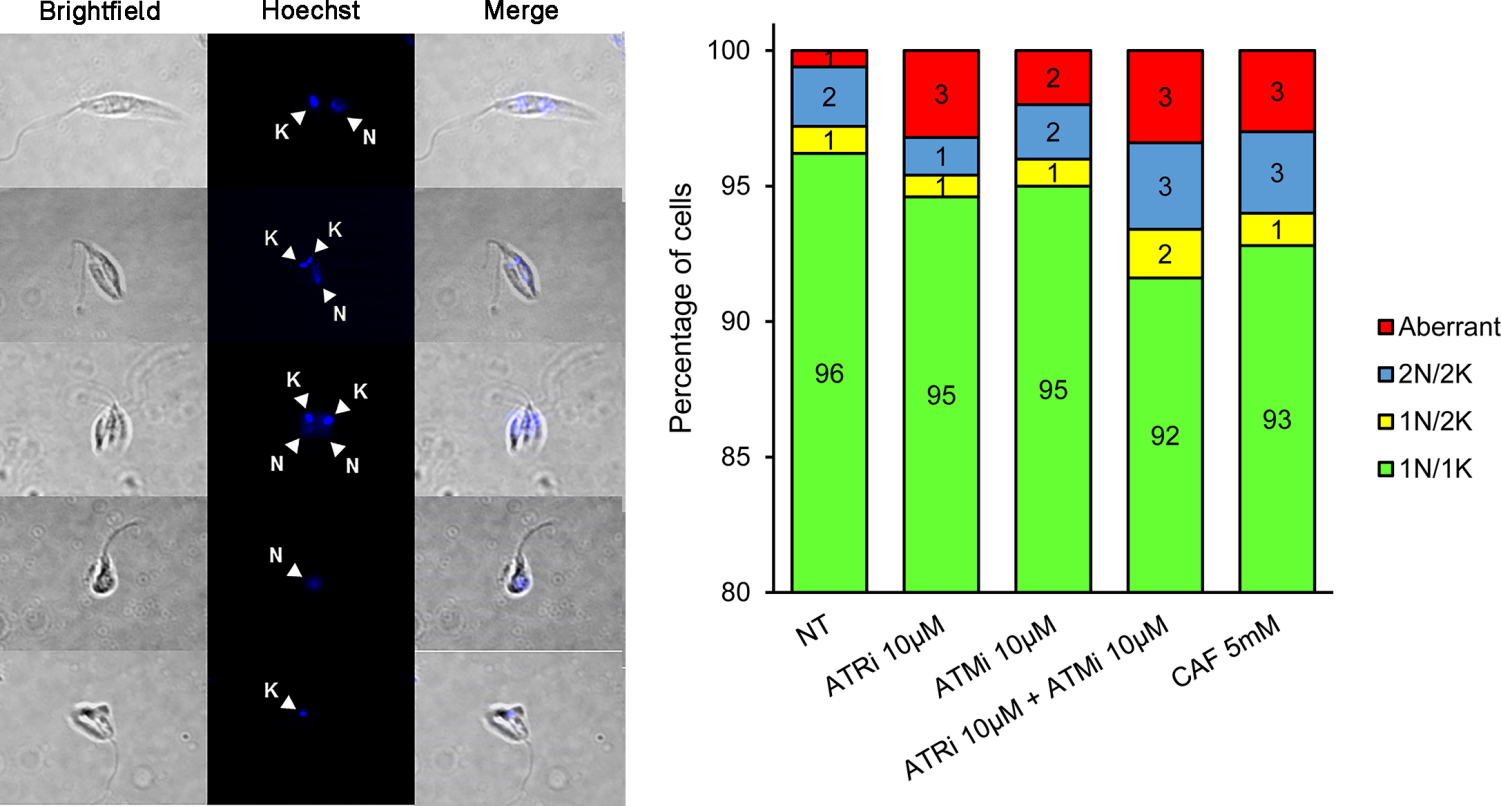
Morphology analysis of *L*. *major* promastigote forms exposed to ATRi, ATMi, and caffeine. *L*. major cells were analyzed by fluorescence microscopy to obtain the number of nuclei and kinetoplasts per cell for each type of treatment: non-treated cells (NT), 10 μM ATRi (VE-821), 10 μM ATMi (KU-55933), combination of 10 μM ATRi and 10 μM ATMi, and 5 mM caffeine (CAF). Five hundred cells of each treatment were analyzed and classified as: 1 nucleus and 1 kinetoplast (1N/1K –green); 1 nucleus and 2 kinetoplasts (1N/2K –yellow); 2 nuclei and 2 kinetoplasts (2N/2K –blue); and cells lacking either nucleus or kinetoplast (“aberrant”–red). The numbers inside the boxes indicate the percentage of cells within each class. Data are representative of at least two independent experiments.

In *L*. *major*, cell division is marked by morphological changes that can be associated with the cell division stage of the parasite [46]. Thus, through fluorescent labeling of nucleic (N) and kinetoplastic (K) DNA, we were able to differentiate 1N/1K, 1N/2K, 2N/2K cells, including aberrant forms lacking either nuclei or kinetoplasts, indicated as 1N/0K or 0N/1K (Fig 7). After 24 hours of treatment with the inhibitors, we observed discrete changes in the proportion of cells, primarily the dose-dependent increase of dysmorphic cells with all treatments (Fig 7, S2 Fig). The major differences were observed once again for caffeine-treated cells, which had the highest proportion of aberrant cells. Cells treated with 20 mM caffeine displayed 26% aberrant cells, with a concomitant decrease in the proportion of 1N/1K cells (from 96% to 69%; Fig 7, S2 Fig). The combinations of ATRi and ATMi resulted in a morphological profile comparable to the observed for 1.25mM and 5mM caffeine treatments (92% to 93% 1N/1K cells, 3% 2N/2K cells and 3% aberrant cells; S2 Fig).

### Human ATR and ATM inhibitors sensitize *L*. *major* cells to oxidative damage

**Fig 8:**
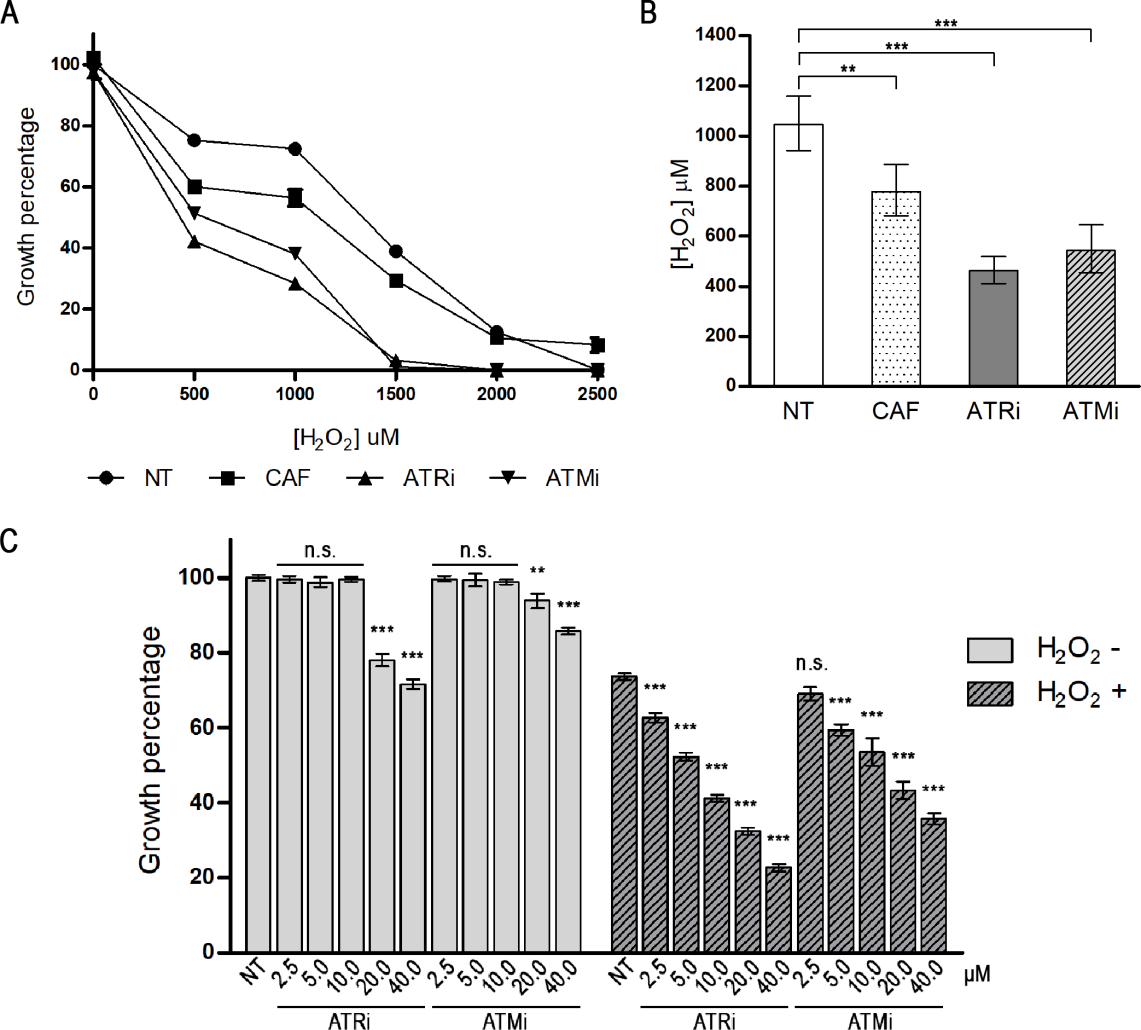
Growth inhibition of *L*. *major* promastigote forms exposed to hydrogen peroxide. (A) Growth percentages of *L*. *major* cells treated with human ATR and ATM inhibitors and exposed to various concentrations of hydrogen peroxide (H_2_O_2_), in comparison with non-treated cells (NT). Cells were treated with 5 mM caffeine (CAF), 10 μM ATRi (VE-821), or 10 μM ATMi (KU-55933) for 1 hour before exposure to H_2_O_2_. Cells were then exposed to 500 μM, 1000 μM, 1500 μM, 2000 μM, or 2500 μM H_2_O_2_ for 20 minutes. Cells were washed for complete removal of drugs and resuspended in culture media. The cells were counted after 72 hours. Percentage of growth is relative to cells non-treated with inhibitors and unexposed to H_2_O_2_. Data represent mean ± SEM of triplicate samples and are representative of three independent experiments. (B) Inhibitory concentrations of H_2_O_2_ for 50% of cell growth (IC_50_) were calculated from the growth curves for each treatment: non-treated cells (NT), 5 mM caffeine (CAF), 10 μM ATRi, or 10 μM ATMi. The H_2_O_2_ inhibitory concentrations recorded for IC_50_ were: NT cells: 1045.0 μM (CI 95%: 941.7–1159.0 μM); caffeine: 777.4 μM (CI 95%: 682.0–886.1 μM); ATRi: 462.1 μM (CI 95%: 411.5–519.0 μM); ATMi: 542.6 μM (CI 95%: 455.7–646.0 μM). Data represent mean ± 95% confidence intervals. Statistical analysis of variance (ANOVA) with post-hoc Dunnett test is displayed: **: p<0.005; ***: p<0.0005. (C) Dose-response evaluation of ATRi and ATMi in unexposed cells (-) and cells exposed to H_2_O_2_ (+). Cells were treated with variable concentrations of inhibitors for 1 hour prior to exposure to 500 μM H_2_O_2_ for 20 minutes. Cells were washed for complete removal of drugs and resuspended in culture media. The cultures were counted after 72 hours. Concentrations of inhibitors are expressed as micromolar (μM). Percentage of growth is relative to cells non-treated to inhibitors and unexposed to H_2_O_2_. Data represents mean ± SEM of three independent experiments. Statistical analysis of variance (ANOVA) with post-hoc Dunnett test is displayed: **: p<0.005; ***: p<0.0005; n.s.: non-significant.

From the data presented previously, we observed that the maintenance of *L*. *major* promastigotes in culture media containing different concentrations of ATRi and ATMi induced small changes in cell proliferation, the cell cycle, and event dynamics that occur during cytokinesis, mainly because these drugs were used alone. Therefore, we decided to test the behavior of cells treated with these inhibitors against oxidative stress, a common challenge that occurs during their life cycle. To compare survival rates with those observed for cells non-treated with protein kinase inhibitors, we treated promastigotes of *L*. *major* with 10 μM ATRi, 10 μM ATMi, or 5 mM caffeine for 1 hour, before exposing the cells to hydrogen peroxide (H_2_O_2_) as a source of oxidative damage. After 72 hours of exposure to H_2_O_2_, we observed a significant reduction in the growth of cells treated with ATRi, ATMi, and caffeine inhibitors compared to that of non-treated cells (p <0.0005, Fig 8A, S3 Fig). We also observed significant reductions in the IC_50_ for H_2_O_2_, from 25.6% for cells treated with 5 mM caffeine, up to 55.8% and 51.9% for ATRi- and ATMi-treated cells, respectively (p <0.005, Fig 8B). These effects were dose-dependent for both inhibitors up to the concentration of 10 μM, since the use of inhibitors above this concentration appeared to cause a non-specific effect, as evaluated for treated cells not exposed to H_2_O_2_ (Fig 8C).

## Discussion

The PIKK proteins are present in a range of organisms, including unicellular fungi such as *S*. *cerevisiae* to more complex organisms such as plants (*A*. *thaliana*) and animals (*C*. *elegans*, *D*. *melanogaster*, *M*. *musculus*, and *H*. *sapiens*). The trypanosomatids, members of the phylum Euglenozoa, are organisms that arose from the earliest eukaryotes, and they retain most of the PIKK proteins [47, 48]. The mTOR protein regulates cell proliferation through the control of processes including mRNA translation, ribosome biogenesis, autophagy and cellular metabolism [49]. ATM acts as the central coordinator of the cellular response to DNA double strand breaks (DSB)[50]. Previous studies also suggest ATM participates in the response to replicative stress, in the mitotic spindle checkpoint and in cytoplasmic pathways, for example, the mobilization of calcium and potassium ions [51–55]. Additionally, ATR is a member of DDR that works during replicative stress by suppressing new origin firing, protecting and reactivating stalled forks, and preventing mitotic anomalies, including chromosome fragmentation [56–58].

ATR and ATM have a similar structural organization, with catalytic domains in the C-terminal portion, flanked by FAT, PRD, and FATC domains [59]. We have demonstrated that, despite the considerable degree of sequence conservation, and size and structure of the C-terminus domains of these PIKK proteins, this region corresponds to a small fraction of the whole protein, from 5.73% to 8.51% of the total amino acids. The remainder corresponds to the large N-terminus, which contains many of the differences that these proteins have in each organism and it contains dozens of HEAT repeats. This motif, consisting of a pair of antiparallel α-helices linked by a flexible loop, forms large chains of super-helical solenoid conformations that serve as a protein-protein interaction interface or for DNA interaction [30, 31, 39, 60, 61]. For example, ATM interacts with Nbs1, a regulator of its activation, through HEAT motifs [62, 63]. ATR interacts with ATRIP through the HEAT repeats in the N-terminus [64]. HEAT repeats also serve as regulatory domains of these proteins, and mutations in this region alter their function [65, 66]. Our data reveals that this region diverges considerably amongst the analyzed organisms, primarily in terms of size, since the trypanosomatids appear to have a larger number of HEAT motifs.

Owing to the high level of conservation of the C-terminus in these proteins, particularly the catalytic domain, we hypothesized that it would be possible to use inhibitory compounds originally developed for human ATR and ATM in this protozoan. VE-821 (ATRi) and KU-55933 (ATMi) are selective inhibitors of human ATR and ATM respectively, with poor association with other off-target kinases [43, 67]. The predicted high affinity of these molecules for the putative *Lmj*ATR and *Lmj*ATM proteins gave us support for testing their effects on *L*. *major* promastigotes. Used alone or in combination, these inhibitory compounds decreased the cellular growth rate, possibly due to G1/S checkpoint arrest as a result of the inhibition of *Lmj*ATR and *Lmj*ATM, which can be observed by the slight accumulation of cells in G1. The highest concentration of ATMi (40 μM) also prevented cells from reaching maximum cell number, possibly by inducing early senescence, since a large reduction in the number of S and G2 cells was observed following 48 hours of treatment. Additionally, the accumulation of cells in G1, and an increase of cells in subG1 was also observed. Combinations of ATRi and ATMi, limited cell proliferation or induced cell death during the first 48 hours, mimicking the inhibitory effect of caffeine on both kinases. Cell cycle arrest in G1 and proliferation rate decrease was also observed in MDA-MB-453 and PC-3 mammary cancer lineages treated with ATMi [68]. Moreover, it has been reported that ATRi could cause an increase in the number of fired replication origins, but with decreased speed of the active forks in RKO cells, consistent with the central role of ATR in DNA replication [69]. Taken together, these data suggest the participation of these kinases in both regular genome replication and cellular proliferation control of *Leishmania* parasites.

The effects on the cell cycle led us to investigate whether there were changes in the cell division kinetics of *L*. *major* when exposed to these inhibitors. In *L*. *major*, cells found in G1 had 1N/1K (1 nucleus and 1 kinetoplast), whereas 2N/2K (2 nuclei and 2 kinetoplasts) represented cells that had undergone division of the nucleus and then going through cytokinesis. The intermediate form of these, 1N/2K, represents cells that are in the process of dividing the newly replicated nucleus in two [46]. Here, we observed minor changes in cell configuration when compared to those in non-treated cells. The clearest change was the appearance of aberrant cells that lacked a nucleus or a kinetoplast. The proportion of aberrant cells increased with the dose of the drug used or a combination of drugs, and this observation was most evident with caffeine. This may indicate that the possible disruption of ATM and ATR kinase-dependent signaling would result in the failure of kinetoplast and/or nucleus division. For instance, in procyclic forms of *T*. *brucei*, inhibition of mitosis by knockdown of various cyclins does not stop the kinetoplast division cycle. The cycle continues with the processes of kinetoplast segregation, cytokinesis, and cell division, giving rise to cells lacking nuclei [70–72]. Therefore, it seems plausible that inhibition of the putative *Lmj*ATR and *Lmj*ATM proteins could interfere with the cycle progression and cell division of *L*. *major*, however further investigation is necessary to confirm these mechanisms.

The effects of ATRi and ATMi on the cell cycle of *L*. *major* promastigote forms maintained in axenic cultures were subtle. This demonstrates that these selective inhibitors do not elicit important effects when applied outside the context of DNA damage. In fact, it has been shown that these inhibitors alone do not produce significant effects on the cell viability of several human tumor lines, in concentrations up to 10 μM for both inhibitors [43, 73]. Therefore, we decided to evaluate the effect of these inhibitors on *L*. *major* exposed to H_2_O_2_. Beyond the proven ability of these inhibitors to potentiate cytotoxic effects of DNA damaging agents in a variety of cancer cell lines, H_2_O_2_-induced oxidative damage could account for many of the challenges that parasites encounter during their life cycle. This is further supported considering the ROS-enriched environment these parasites have within the parasitophore vacuole [19, 74–80]. ROS has a key role in *Leishmania* differentiation from the promastigote to the amastigote form. These parasites can rely on DNA repair proteins to protect them from ROS-induced toxic effects, whether they are derived from macrophage activity or leishmanicidal drugs, such as amphotericin B and antimony (V) sodium gluconate [81–83]. This demonstrates the participation of critical pathways regulating genomic stability in *Leishmania*, which motivated this investigation.

Evidence suggests that ATM-deficient mammalian cells or cells derived from patients with ataxia-telangiectasia syndrome (A-T) present with high ROS levels and hypersensitivity to agents that cause oxidative damage [84–86]. Chk1 is phosphorylated by ATR after DNA damage or replication fork arrest caused by H_2_O_2_ in human cells or *Xenopus* egg extracts, which demonstrates the quick response of this kinase to oxidative damage [87, 88]. We found that *L*. *major* is highly resistant to the effects of H_2_O_2_. However, cellular survival changes following the addition of ATR and ATM inhibitors. Whilst caffeine reduced the IC_50_ by 25% of that in non-treated cells, ATRi and ATMi reduced IC_50_ values to less than half. Further, at H_2_O_2_ concentrations of approximately 1.5mM, 3% or less of cells remained in the cultures treated with these inhibitors. We have shown that this effect is dose-dependent up to 10 μM of the inhibitors, which strongly suggest they could be acting in a specific manner. These data could indicate that specific inactivation of ATR and ATM by ATRi and ATMi interferes with *L*. *major* tolerance to damage caused by H_2_O_2_-induced ROS. Bagley et al. [89] reported that ATMi also inhibited proliferation and induced apoptosis of T murine cells after stimulation with anti-CD3 and anti-CD28, features that were reverted by the addition of an antioxidant agent. In contrast, Biskup et al. [75] did not observe any synergistic effect of VE-821 or VE-822, an analogue of the former, on cytotoxicity caused by H_2_O_2_ in a cutaneous T-cell lymphoma lineage. Such differences could rely on how ATR and ATM, and other kinases, behave when presented with oxidative damage. The different actions of these PIKK proteins within each cell type reflect the unique signaling pathways present in these cells and trypanosomatids in general.

In this work, we demonstrated evidence for putative homologs of the ATR and ATM genes in *Leishmania major*. These PIKK proteins are conserved in the protozoan genome, mainly in the carboxy-terminal portion, which contains the catalytic domain. Evolutionarily, they are related to ATR and ATM proteins of other organisms, as well as mTOR and DNA-PKcs. Owing to the conservation of the residues and the structure of the catalytic domain, we could infer that inhibitory compounds for human ATR and ATM could also work in *L*. *major*. Used separately or in combination, the inhibitors ATRi and ATMi induced minor changes in cell proliferation, cycle and morphology. However, when associated with a DNA-damaging agent, in this case hydrogen peroxide, these inhibitors could hypersensitize *L*. *major* promastigotes. This suggests their potential capacity to inhibit the DDR pathways in this parasite, which may help in the elucidation of the pathways involved in the genomic stability of *Leishmania*. Leishmaniases, diseases caused by these protozoa, are successfully treated in some patients. However, as treatment is marked by toxicity, high cost, long duration with failures and relapses, administration difficulties, and increasing drug resistance, not all patients are cured of disease [90]. A similar scenario is observed for cancer therapy, for which VE-821, KU-55933, their analogues and many other specific kinase inhibitors have been created, following an expectation that these compounds could render cancer cells more susceptible to available chemo- and radiotherapeutics. Therefore, it is possible that alternative treatments of the leishmaniases could arise from drug repurposing strategies. In fact, the development of new therapeutic modalities that can provide an effective, safe, low-cost, and short-term treatment is essential. The possibility of using essential targets for DNA maintenance to potentiate the effects of leishmanicidal drugs may form the bridge to this objective.

## Supporting information

S1 Table PIKK domain sequences used to construct the radial cladogram. Selected *L*. *major* sequences (*Lmj*F.32.1460, *Lmj*F.02.0120, *Lmj*F.36.6320, *Lmj*F.34.4530, *Lmj*F.36.2940) were used as queries in a PSI-BLAST search in the non-redundant protein sequences databank of six other *Leishmania* species (*Leishmania infantum*, *Leishmania donovani*, *Leishmania mexicana*, *Leishmania guyanensis*, *Leishmania panamensis* and *Leishmania braziliensis*), and trypanosomatids (genus *Leptomonas* and *Trypanosoma*), as well as model organisms (*Homo sapiens*, *Mus musculus*, *Saccharomyces cerevisiae*, *Caenorhabditis elegans*, *Arabdopsis thaliana*, *Drosophila melanogaster*). The selected sequences were analyzed with CD-Search to delimit the catalytic domain of each PIKK. (a) Protein size in residues. (b) Range of residue positions for the rescued proteins PIKK domain, as predicted by CD-Search. (c) Only partial sequence was available from GenBank.
(PDF)

S2 Table Percentages of identity among selected sequences of ATR and ATM. Percentages of identity for sequences of several ATR and ATM homologs when aligned using CLUSTAL Omega. Sequences were first submitted to CD-Search for prediction of their catalytic domain constituent amino acids, as seen in S1 Table. Then, the sequences were separated in full length, amino-terminal (N-terminal) and carboxy-terminal (C-terminal) for alignment. N-terminal ranges from the first amino acid of the protein to the first amino acid of the catalytic domain; C-terminal ranges from the first amino acid of the catalytic domain to the final residue of the protein.
(PDF)

S3 Table Percentages of cell cycle stages of *Leishmania major* promastigotes treated with ATR and ATM inhibitors. Log phase *L*. *major* cells were maintained in culture media containing caffeine (CAF– 1.25mM, 5mM and 20mM), ATRi (2.5μM, 10μM, 40μM), ATMi (2.5μM, 10μM, 40μM), or combinations of ATRi and ATMi (10μM ATRi + 10μM ATMi, 20μM ATRi + 20μM ATMi), and were compared to non-treated control (NT). Samples (0.5 to 2 × 10^7^ cells) were collected shortly after the inoculum (0h) and after 24 h, 48 h, and 72 h of incubation with the inhibitors. The proportions of cells at each stage of the cell cycle (<G1, G1, S, G2 and >G2) were analyzed using the chi-squared test. (a) Chi-squared test obtained p-value. (b) Owing to the high cell mortality, the total of cells analyzed was reduced for treatment with 20 mM caffeine.
(PDF)

S1 Fig Complete cell cycle analysis of *L*. *major* promastigote forms exposed to ATRi, ATMi and caffeine. Log phase *L*. *major* cells were maintained in culture media containing caffeine (CAF– 1.25mM, 5mM and 20mM), ATRi (2.5μM, 10μM, 40μM), ATMi (2.5μM, 10μM, 40μM), or combinations of ATRi and ATMi (10μM ATRi + 10μM ATMi, 20μM ATRi + 20μM ATMi), and were compared to non-treated control (NT). Samples (0.5 to 2 × 10^7^ cells) were collected shortly after the inoculum (0h) and after 24 h, 48 h, and 72 h of incubation with the inhibitors. Treated cells are displayed as red curves whereas non-treated cells are displayed as blue curves.
(TIF)

S2 Fig Complete morphology analysis of *L*. *major* promastigote forms exposed to ATRi, ATMi and caffeine. *L*. *major* cell promastigotes were analyzed by fluorescence microscopy to obtain both the number of nuclei and kinetoplasts per cell treated with caffeine (CAF– 1.25mM, 5mM and 20mM), ATRi (2.5μM, 10μM, 40μM), ATMi (2.5μM, 10μM, 40μM), or combinations of ATRi and ATMi (10μM ATRi + 10μM ATMi, 20μM ATRi + 20μM ATMi), in comparison with non-treated controls (NT). Five hundred cells of each treatment were analyzed and classified as: 1 nucleus and 1 kinetoplast (1N/1K –green); 1 nucleus and 2 kinetoplasts (1N/2K –yellow); 2 nuclei and 2 kinetoplasts (2N/2K –blue); and cells lacking either nucleus or kinetoplast (“aberrant”–red). Numbers inside the boxes display the percentage of cells within each class. Data are representative of at least two independent experiments.
(TIF)

S3 Fig Best fit curves of nonlinear regression for the calculation of IC_50_. Data from three independent experiments were used to construct the best fit curves for the nonlinear regression calculation of IC_50_ for H_2_O_2_ for cells treated with 10 μM ATRi (VE-821 –green), 10 μM ATMi (KU-55933 –yellow), or 5 mM caffeine (CAF–red), in comparison with cells non-treated by inhibitors (NT–blue). The concentrations of H_2_O_2_ used are expressed as log of the concentration in micromolar. The goodness of fit is indicated as R-squared values displayed for each curve.
(TIF)
