# Supplementary material for: Selective human inhibitors of ATR and ATM render Leishmania major promastigotes sensitive to oxidative damage
Source: PLoS One. 2018 Sep 28;13(9):e0205033. doi: 10.1371/journal.pone.0205033 (PMC6161909; doi:10.1371/journal.pone.0205033)
Supplement: S1 Table — Selected L. major sequences (LmjF.32.1460, LmjF.02.0120, LmjF.36.6320, LmjF.34.4530, LmjF.36.2940) were used as queries in a PSI-BLAST search in the non-redundant protein sequences databank of six other Leishmania species (Leishmania infantum, Leishmania donovani, Leishmania mexicana, Leishmania guyanensis, Leishmania panamensis and Leishmania braziliensis), and trypanosomatids (genus Leptomonas and Trypanosoma), as well as model organisms (Homo sapiens, Mus musculus, Saccharomyces cerevisiae, Caenorhabditis elegans, Arabdopsis thaliana, Drosophila melanogaster). The selected sequences were analyzed with CD-Search to delimit the catalytic domain of each PIKK. (a) Protein size in residues. (b) Range of residue positions for the rescued proteins PIKK domain, as predicted by CD-Search. (c) Only partial sequence was available from GenBank. (PDF) [file pone.0205033.s001.pdf]

Organism	GenBank accession number	Protein size[a]	Domain size[a] (range[b])
**ATR**			
Leishmania major	XP_001685462.1	3207	273 (2863-3135)
Leishmania infantum	XP_001467832.1	3211	273 (2867-3139)
Leishmania donovani	XP_003863568.1	3211	273 (2867-3139)
Leishmania mexicana	XP_003877990.1	3211	273 (2867-3139)
Leishmania guyanensis	CCM18220.1	3207	275 (2863-3137)
Leishmania panamensis	XP_010701840.1	3207	275 (2863-3137)
Leishmania braziliensis	XP_001567518.2	3207	275 (2863-3137)
Leptomonas seymouri	KPI90816.1	3216	275 (2872-3146)
Leptomonas pyrrhocoris	XP_015663127.1	3225	275 (2881-3155)
Trypanosoma grayi	XP_009306375.1	2830	275 (2486-2760)
Trypanosoma brucei brucei	XP_829526.1	2860	275 (2516-2790)
Trypanosoma cruzi	ESS66299.1	2874	275 (2530-2804)
Trypanosoma cruzi marinkellei	EKF33229.1	2869	275 (2525-2799)
Trypanosoma vivax	CCC54057.1	2883	275 (2539-2813)
Trypanosoma rangeli	ESL05845.1	2318	275 (1974-2248)
Homo sapiens	NP_001175.2	2644	275 (2293-2567)
Mus musculus	NP_063917.1	2641	275 (2290-2564)
Saccharomyces cerevisiae	NP_009694.3	2368	270 (2046-2315)
Caenorhabditis elegans	NP_505487.3	2531	270 (2189-2458)
Arabidopsis thaliana	NP_001332644.1	2703	276 (2364-2639)
Drosophila melanogaster	NP_523369.2	2517	268 (2181-2448)
**ATM**			
Leishmania major	XP_003721617.1	4905	281 (4537-4817)
Leishmania infantum	XP_001462707.1	4906	281 (4533-4813)
Leishmania donovani	XP_003857906.1	4905	281 (4532-4812)
Leishmania mexicana	XP_003871615.1	4922	281 (4556-4836)
Leishmania braziliensis	XP_001561526.2	4924	281 (4548-4828)
Leishmania guyanensis	CCM12551.1	4450[c]	281 (4074-4354)
Leishmania panamensis	XP_010698154.1	4929	281 (4553-4833)
Leptomonas pyrrhocoris	XP_015653282.1	4873	281 (4486-4766)
Leptomonas seymouri	KPI85348.1	4881	279 (4502-4780)
Trypanosoma cruzi	ESS63096.1	4232	288 (3845-4132)
Trypanosoma brucei brucei	XP_951535.1	4277	279 (3902-4180)
Trypanosoma rangeli	ESL05811.1	4178	297 (3774-4070)
Trypanosoma vivax	CCC46616.1	4098[c]	279 (3737-4015)
Homo sapiens	NP_000042.3	3056	280 (2683-2962)
Mus musculus	NP_031525.2	3066	280 (2693-2972)
Saccharomyces cerevisiae	NP_009465.2	2787	275 (2430-2704)
Drosophila melanogaster	NP_001036712.1	2767	281 (2416-2696)
Arabidopsis thaliana	NP_001326745.1	2989	278 (2629-2906)
Caenorhabditis elegans	NP_001293214.1	2378	273 (2041-2313)
**mTOR**			
Leishmania major	XP_001686560.1	2438	285 (2033-2317)
Leishmania infantum	XP_001468774.1	2438	285 (2033-2317)
Leishmania donovani	XP_003864576.1	2437	285 (2032-2316)
Leishmania mexicana	XP_003878997.1	2438	285 (2033-2317)
Leishmania guyanensis	CCM15140.1	2437	285 (2032-2316)
Leishmania panamensis	XP_010698613.1	2437	285 (2032-2316)
Leishmania braziliensis	XP_001564679.2	2437	282 (2035-2316)
Leptomonas seymouri	KPI85294.1	2438	282 (2036-2317)
Leptomonas pyrrhocoris	XP_015661018.1	2438	283 (2035-2317)
Trypanosoma cruzi	ESS68725.1	2434	285 (2031-2315)
Trypanosoma cruzi marinkellei	EKF33676.1	2434	285 (2031-2315)
Trypanosoma rangeli	ESL05666.1	2434	285 (2031-2315)
Trypanosoma grayi	XP_009310318.1	2437	285 (2033-2317)
Trypanosoma vivax	CCC47348.1	2360[c]	284 (1957-2240)
Trypanosoma brucei brucei	XP_844192.1	2412	284 (2009-2292)
Homo sapiens	NP_004949.1	2549	279 (2153-2431)
Mus musculus	NP_064393.2	2549	279 (2153-2431)
Saccharomyces cerevisiae	NP_012719.2	2474	277 (2094-2370)
Drosophila melanogaster	NP_524891.1	2470	279 (2074-2352)
Arabidopsis thaliana	NP_175425.2	2481	279 (2062-2340)
Caenorhabditis elegans	NP_491549.2	2695	280 (2327-2606)
**DNA-PKcs**			
Leishmania major	XP_001686873.1	4183	304 (3799-4102)
Leishmania donovani	XP_003865424.1	4179	304 (3795-4098)
Leishmania infantum	XP_001469701.1	4178	304 (3794-4097)
Leishmania mexicana	XP_003874646.1	4175	304 (3791-4094)
Leishmania braziliensis	XP_001568935.2	4193	267 (3809-4075)
Leishmania guyanensis	CCM19640.1	4193	267 (3809-4075)
Leishmania panamensis	XP_010703238.1	4193	267 (3809-4075)
Homo sapiens	NP_008835.5	4128	297 (3719-4015)
Mus musculus	NP_035289.2	4128	296 (3719-4014)
