# Supplementary material for: Selective human inhibitors of ATR and ATM render Leishmania major promastigotes sensitive to oxidative damage
Source: PLoS One. 2018 Sep 28;13(9):e0205033. doi: 10.1371/journal.pone.0205033 (PMC6161909; doi:10.1371/journal.pone.0205033)
Supplement: S2 Table — Percentages of identity for sequences of several ATR and ATM homologs when aligned using CLUSTAL Omega. Sequences were first submitted to CD-Search for prediction of their catalytic domain constituent amino acids, as seen in S1 Table. Then, the sequences were separated in full length, amino-terminal (N-terminal) and carboxy-terminal (C-terminal) for alignment. N-terminal ranges from the first amino acid of the protein to the first amino acid of the catalytic domain; C-terminal ranges from the first amino acid of the catalytic domain to the final residue of the protein. (PDF) [file pone.0205033.s002.pdf]

**ATR**	*L. major*			*L. infantum*			*L. braziliensis*			*T. brucei*			*T. cruzi*			*H. sapiens*			*S. cerevisiae*		
	Full	N-terminal	C-terminal	Full	N-terminal	C-terminal	Full	N-terminal	C-terminal	Full	N-terminal	C-terminal	Full	N-terminal	C-terminal	Full	N-terminal	C-terminal	Full	N-terminal	C-terminal
L. major	100.00	100.00	100.00	93.70	93.12	98.55	80.56	78.59	96.81	31.73	26.80	63.77	32.14	27.89	61.45	20.85	18.49	37.35	16.56	14.06	31.27
L. infantum	93.70	93.12	98.55	100.00	100.00	100.00	80.57	78.57	97.10	32.31	27.71	64.35	32.29	28.42	61.74	20.78	18.76	37.65	16.58	14.21	31.27
L. braziliensis	80.56	78.59	96.81	80.57	78.57	97.10	100.00	100.00	100.00	31.98	27.63	62.61	31.86	27.93	60.87	20.47	17.94	36.76	16.31	13.67	32.20
T. brucei	31.73	26.80	63.77	32.31	27.71	64.35	31.98	27.63	62.61	100.00	100.00	100.00	48.72	44.55	78.55	20.24	16.80	40.59	17.27	14.23	32.20
T. cruzi	32.14	27.89	61.45	32.29	28.42	61.74	31.86	27.93	60.87	48.72	44.55	78.55	100.00	100.00	100.00	20.79	17.44	38.82	17.38	14.55	33.13
H. sapiens	20.85	18.49	37.35	20.78	18.76	37.65	20.47	17.94	36.76	20.24	16.80	40.59	20.79	17.44	38.82	100.00	100.00	100.00	23.58	20.05	43.65
S. cerevisiae	16.56	14.06	31.27	16.58	14.21	31.27	16.31	13.67	32.20	17.27	14.23	32.20	17.38	14.55	33.13	23.58	20.05	43.65	100.00	100.00	100.00

**ATM**	*L. major*			*L. infantum*			*L. braziliensis*			*T. brucei*			*T. cruzi*			*H. sapiens*			*S. cerevisiae*		
	Full	N-terminal	C-terminal	Full	N-terminal	C-terminal	Full	N-terminal	C-terminal	Full	N-terminal	C-terminal	Full	N-terminal	C-terminal	Full	N-terminal	C-terminal	Full	N-terminal	C-terminal
L. major	100.00	100.00	100.00	90.02	89.38	97.83	72.98	71.72	88.35	29.66	27.25	53.68	31.01	28.45	55.56	19.97	17.68	39.72	17.46	15.02	36.76
L. infantum	90.02	89.38	97.83	100.00	100.00	100.00	72.91	71.80	86.36	29.43	27.04	53.23	31.07	28.54	55.35	19.93	17.43	39.39	17.18	14.15	36.34
L. braziliensis	72.98	71.72	88.35	72.91	71.80	86.36	100.00	100.00	100.00	29.49	26.92	53.60	30.87	28.40	54.38	20.42	17.64	39.45	18.10	15.48	36.60
T. brucei	29.66	27.25	53.68	29.43	27.04	53.23	29.49	26.92	53.60	100.00	100.00	100.00	48.09	45.54	73.14	20.96	15.84	42.62	17.46	13.34	38.44
T. cruzi	31.01	28.45	55.56	31.07	28.54	55.35	30.87	28.40	54.38	48.09	45.54	73.14	100.00	100.00	100.00	21.15	16.91	44.99	17.45	12.63	39.60
H. sapiens	19.97	17.68	39.72	19.93	17.43	39.39	20.42	17.64	39.45	20.96	15.84	42.62	21.15	16.91	44.99	100.00	100.00	100.00	22.61	19.20	43.68
S. cerevisiae	17.46	15.02	36.76	17.18	14.15	36.34	18.10	15.48	36.60	17.46	13.34	38.44	17.45	12.63	39.60	22.61	19.20	43.68	100.00	100.00	100.00
