# Supplementary material for: Selective human inhibitors of ATR and ATM render Leishmania major promastigotes sensitive to oxidative damage
Source: PLoS One. 2018 Sep 28;13(9):e0205033. doi: 10.1371/journal.pone.0205033 (PMC6161909; doi:10.1371/journal.pone.0205033)
Supplement: S3 Table — Log phase L. major cells were maintained in culture media containing caffeine (CAF– 1.25mM, 5mM and 20mM), ATRi (2.5μM, 10μM, 40μM), ATMi (2.5μM, 10μM, 40μM), or combinations of ATRi and ATMi (10μM ATRi + 10μM ATMi, 20μM ATRi + 20μM ATMi), and were compared to non-treated control (NT). Samples (0.5 to 2 × 10^7^ cells) were collected shortly after the inoculum (0h) and after 24 h, 48 h, and 72 h of incubation with the inhibitors. The proportions of cells at each stage of the cell cycle (<G1, G1, S, G2 and >G2) were analyzed using the chi-squared test. (a) Chi-squared test obtained p-value. (b) Owing to the high cell mortality, the total of cells analyzed was reduced for treatment with 20 mM caffeine. (PDF) [file pone.0205033.s003.pdf]

| Treatment | Cell cycle stage | | | | | | | |
|---|---|---|---|---|---|---|---|---|
| | <G1 | G1 | S | G2 | >G2 | Ratio 4N/2N | Total of cells analyzed | p value[a] |
| **0h after treatment** | | | | | | | | |
| NT | 10.80 | 36.20 | 24.80 | 27.90 | 0.29 | 1.94 | 8180 | |
| ATRi 2.5 µM | 11.90 | 31.20 | 29.20 | 24.70 | 3.00 | 1.96 | 8677 | |
| ATRi 10 µM | 12.70 | 37.30 | 23.00 | 25.90 | 0.99 | 1.95 | 8645 | |
| ATRi 40 µM | 12.20 | 31.30 | 34.00 | 19.40 | 3.11 | 2.01 | 7531 | |
| ATMi 2.5 µM | 9.09 | 28.50 | 33.10 | 25.90 | 3.34 | 1.97 | 8556 | |
| ATMi 10 µM | 11.00 | 31.90 | 28.70 | 27.90 | 0.54 | 1.95 | 8847 | 0.7967 |
| ATMi 40 µM | 13.70 | 35.20 | 21.50 | 29.60 | 0.00 | 1.95 | 8864 | |
| ATRi 10 µM + ATMi 10 µM | 9.95 | 32.60 | 30.60 | 22.50 | 4.44 | 1.96 | 8640 | |
| ATRi 20 µM + ATMi 20 µM | 11.70 | 35.20 | 27.10 | 24.00 | 1.98 | 1.99 | 8653 | |
| CAF 1.25 mM | 13.10 | 27.00 | 29.70 | 29.70 | 0.56 | 1.95 | 8079 | |
| CAF 5 mM | 10.70 | 27.70 | 32.70 | 25.40 | 3.50 | 1.95 | 8816 | |
| CAF 20 mM | 5.89 | 34.50 | 23.50 | 36.10 | 0.00 | 1.95 | 8639 | |
| **24h after treatment** | | | | | | | | |
| NT | 11.70 | 35.60 | 25.90 | 26.10 | 0.67 | 1.95 | 7637 | |
| ATRi 2.5 µM | 13.60 | 37.20 | 27.80 | 18.30 | 3.00 | 1.95 | 8600 | |
| ATRi 10 µM | 16.30 | 37.90 | 28.10 | 15.40 | 2.28 | 1.98 | 8128 | |
| ATRi 40 µM | 15.70 | 40.20 | 24.90 | 18.10 | 1.07 | 1.95 | 7958 | |
| ATMi 2.5 µM | 12.70 | 32.70 | 32.30 | 18.70 | 3.58 | 1.94 | 8636 | |
| ATMi 10 µM | 12.70 | 32.70 | 32.30 | 18.70 | 3.58 | 1.98 | 8636 | <0.0001 |
| ATMi 40 µM | 12.90 | 47.90 | 21.50 | 15.80 | 1.85 | 1.95 | 8302 | |
| ATRi 10 µM + ATMi 10 µM | 16.70 | 42.60 | 25.20 | 14.80 | 0.59 | 1.96 | 8319 | |
| ATRi 20 µM + ATMi 20 µM | 17.00 | 45.50 | 21.60 | 15.00 | 0.84 | 1.96 | 8336 | |
| CAF 1.25 mM | 13.10 | 38.60 | 27.20 | 19.70 | 1.44 | 1.96 | 8735 | |
| CAF 5 mM | 20.00 | 43.20 | 23.10 | 12.10 | 1.63 | 1.95 | 8036 | |
| CAF 20 mM | 7.02 | 23.20 | 16.00 | 52.30 | 1.51 | 1.95 | 5951[b] | |
| **48h after treatment** | | | | | | | | |
| NT | 13.10 | 32.60 | 30.40 | 20.90 | 2.94 | 1.98 | 8370 | |
| ATRi 2.5 µM | 16.70 | 38.20 | 27.00 | 17.20 | 0.84 | 1.99 | 8613 | |
| ATRi 10 µM | 17.80 | 39.30 | 24.60 | 14.80 | 3.51 | 1.95 | 8632 | |
| ATRi 40 µM | 16.30 | 43.40 | 24.40 | 15.60 | 0.26 | 1.97 | 8209 | |
| ATMi 2.5 µM | 11.90 | 37.40 | 27.40 | 20.50 | 2.77 | 1.98 | 8765 | |
| ATMi 10 µM | 14.20 | 39.60 | 26.00 | 18.40 | 1.86 | 1.95 | 8754 | 0.733 |
| ATMi 40 µM | 17.60 | 48.30 | 19.40 | 14.60 | 0.13 | 2.00 | 7729 | |
| ATRi 10 µM + ATMi 10 µM | 16.30 | 43.70 | 25.60 | 14.20 | 0.26 | 2.02 | 8391 | |
| ATRi 20 µM + ATMi 20 µM | 17.40 | 46.80 | 22.40 | 13.30 | 0.12 | 2.01 | 8266 | |
| CAF 1.25 mM | 12.80 | 44.20 | 23.10 | 18.60 | 1.38 | 1.96 | 8672 | |
| CAF 5 mM | 21.10 | 40.90 | 25.70 | 10.10 | 2.25 | 1.95 | 8117 | |
| **72h after treatment** | | | | | | | | |
| NT | 15.80 | 37.20 | 22.90 | 19.90 | 4.22 | 1.95 | 8661 | |
| ATRi 2.5 µM | 18.60 | 39.60 | 26.00 | 14.20 | 1.64 | 2.02 | 8422 | |
| ATRi 10 µM | 21.50 | 45.60 | 20.10 | 11.40 | 1.30 | 1.98 | 8207 | 0.4053 |
| ATRi 40 µM | 18.00 | 51.70 | 16.70 | 13.50 | 0.18 | 2.04 | 7993 | |
| ATMi 2.5 µM | 15.40 | 44.70 | 18.10 | 19.50 | 2.31 | 1.95 | 8488 | |

| | | | | | | | |
|---|---|---|---|---|---|---|---|
| ATMi 10 μM | 17.20 | 34.80 | 27.70 | 15.30 | 4.96 | 1.96 | 8173 |
| ATMi 40 μM | 17.20 | 53.70 | 14.40 | 14.30 | 0.42 | 1.97 | 7887 |
| ATRi 10 μM + ATMi 10 μM | 17.00 | 44.60 | 22.20 | 15.80 | 0.42 | 2.02 | 8162 |
| ATRi 20 μM + ATMi 20 μM | 18.40 | 45.90 | 20.40 | 14.60 | 0.74 | 1.96 | 8415 |
| CAF 1.25 mM | 17.50 | 43.30 | 19.80 | 18.00 | 1.41 | 1.96 | 8446 |
| CAF 5 mM | 17.90 | 48.40 | 19.50 | 14.00 | 0.26 | 1.95 | 7823 |
