# Supplementary figures and images for: Selective human inhibitors of ATR and ATM render Leishmania major promastigotes sensitive to oxidative damage
Source: PLoS One. 2018 Sep 28;13(9):e0205033. doi: 10.1371/journal.pone.0205033 (PMC6161909; doi:10.1371/journal.pone.0205033)

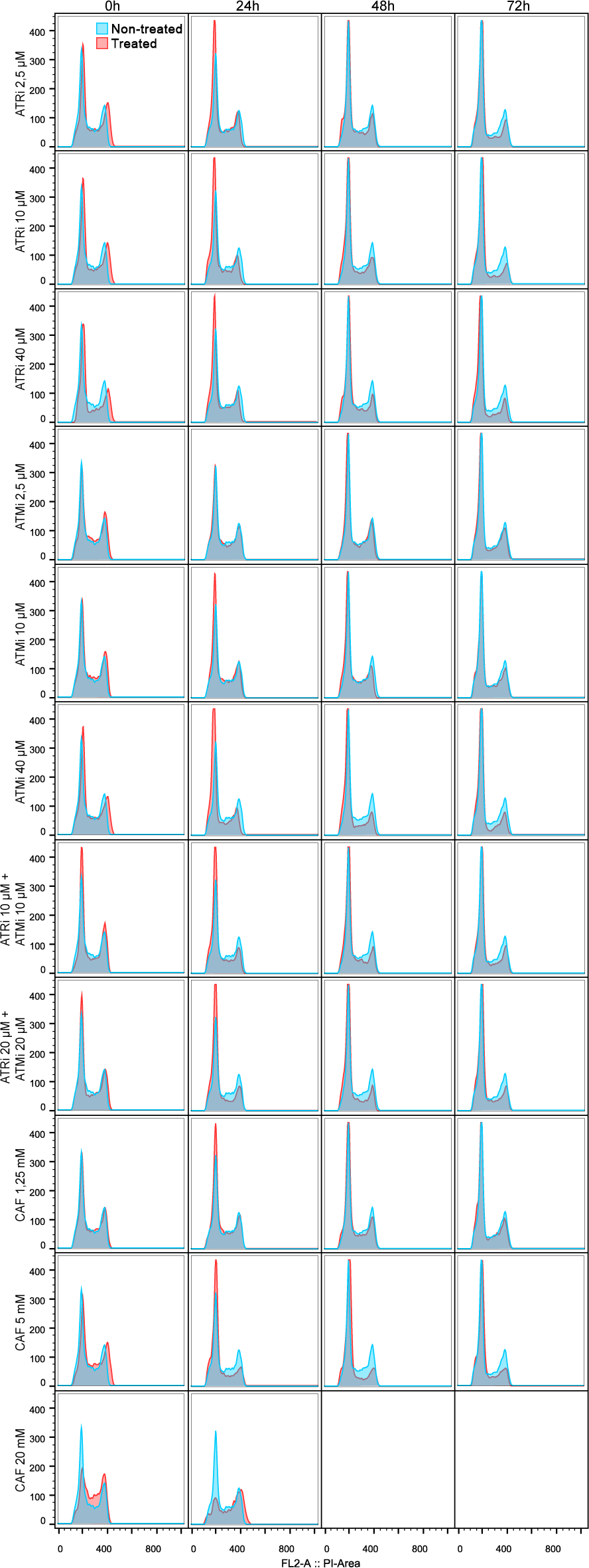

Supplement: S1 Fig — Log phase L. major cells were maintained in culture media containing caffeine (CAF– 1.25mM, 5mM and 20mM), ATRi (2.5μM, 10μM, 40μM), ATMi (2.5μM, 10μM, 40μM), or combinations of ATRi and ATMi (10μM ATRi + 10μM ATMi, 20μM ATRi + 20μM ATMi), and were compared to non-treated control (NT). Samples (0.5 to 2 × 10^7^ cells) were collected shortly after the inoculum (0h) and after 24 h, 48 h, and 72 h of incubation with the inhibitors. Treated cells are displayed as red curves whereas non-treated cells are displayed as blue curves. (TIF) [file pone.0205033.s004.tif]

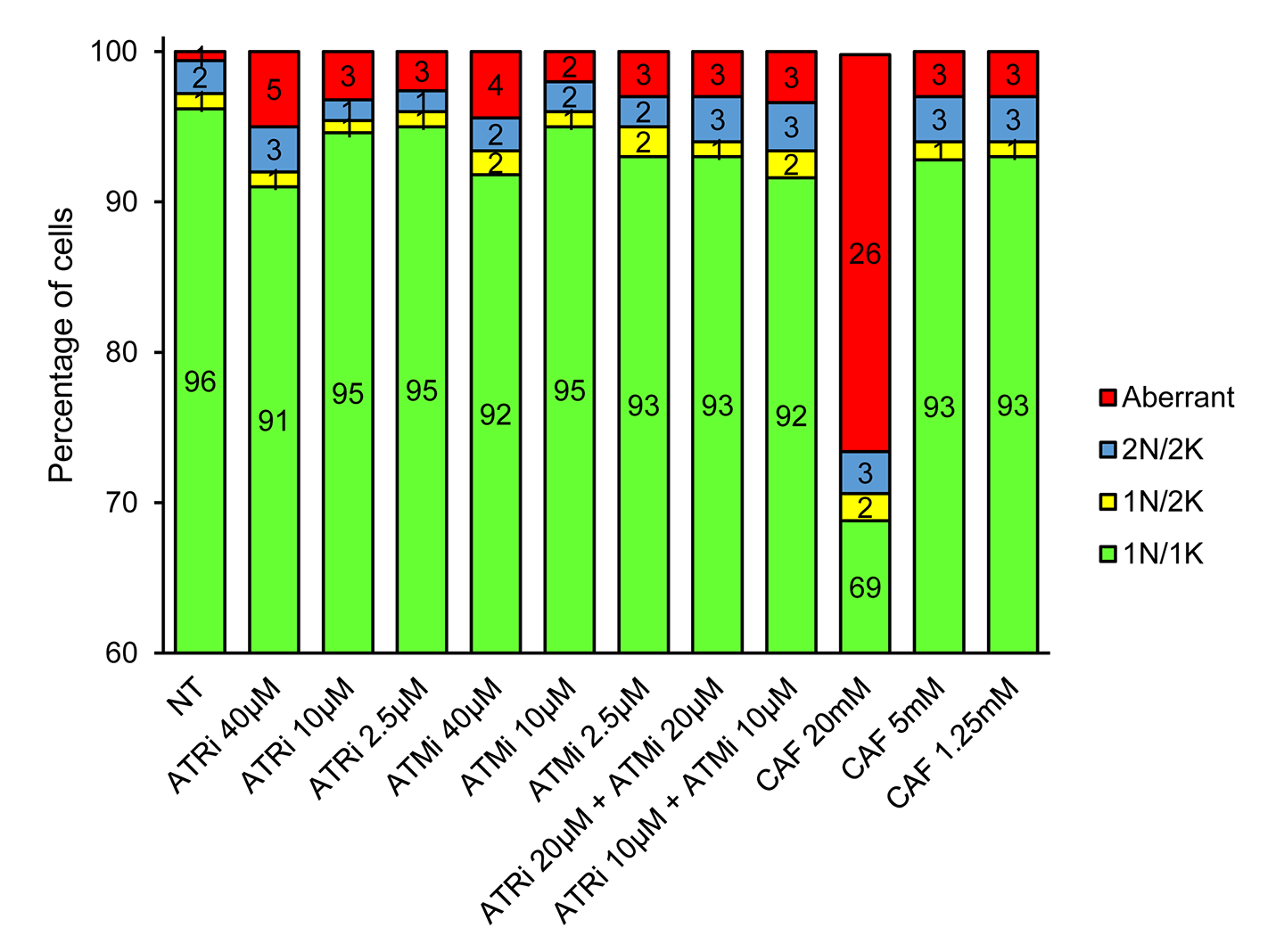

Supplement: S2 Fig — L. major cell promastigotes were analyzed by fluorescence microscopy to obtain both the number of nuclei and kinetoplasts per cell treated with caffeine (CAF– 1.25mM, 5mM and 20mM), ATRi (2.5μM, 10μM, 40μM), ATMi (2.5μM, 10μM, 40μM), or combinations of ATRi and ATMi (10μM ATRi + 10μM ATMi, 20μM ATRi + 20μM ATMi), in comparison with non-treated controls (NT). Five hundred cells of each treatment were analyzed and classified as: 1 nucleus and 1 kinetoplast (1N/1K –green); 1 nucleus and 2 kinetoplasts (1N/2K –yellow); 2 nuclei and 2 kinetoplasts (2N/2K –blue); and cells lacking either nucleus or kinetoplast (“aberrant”–red). Numbers inside the boxes display the percentage of cells within each class. Data are representative of at least two independent experiments. (TIF) [file pone.0205033.s005.tif]

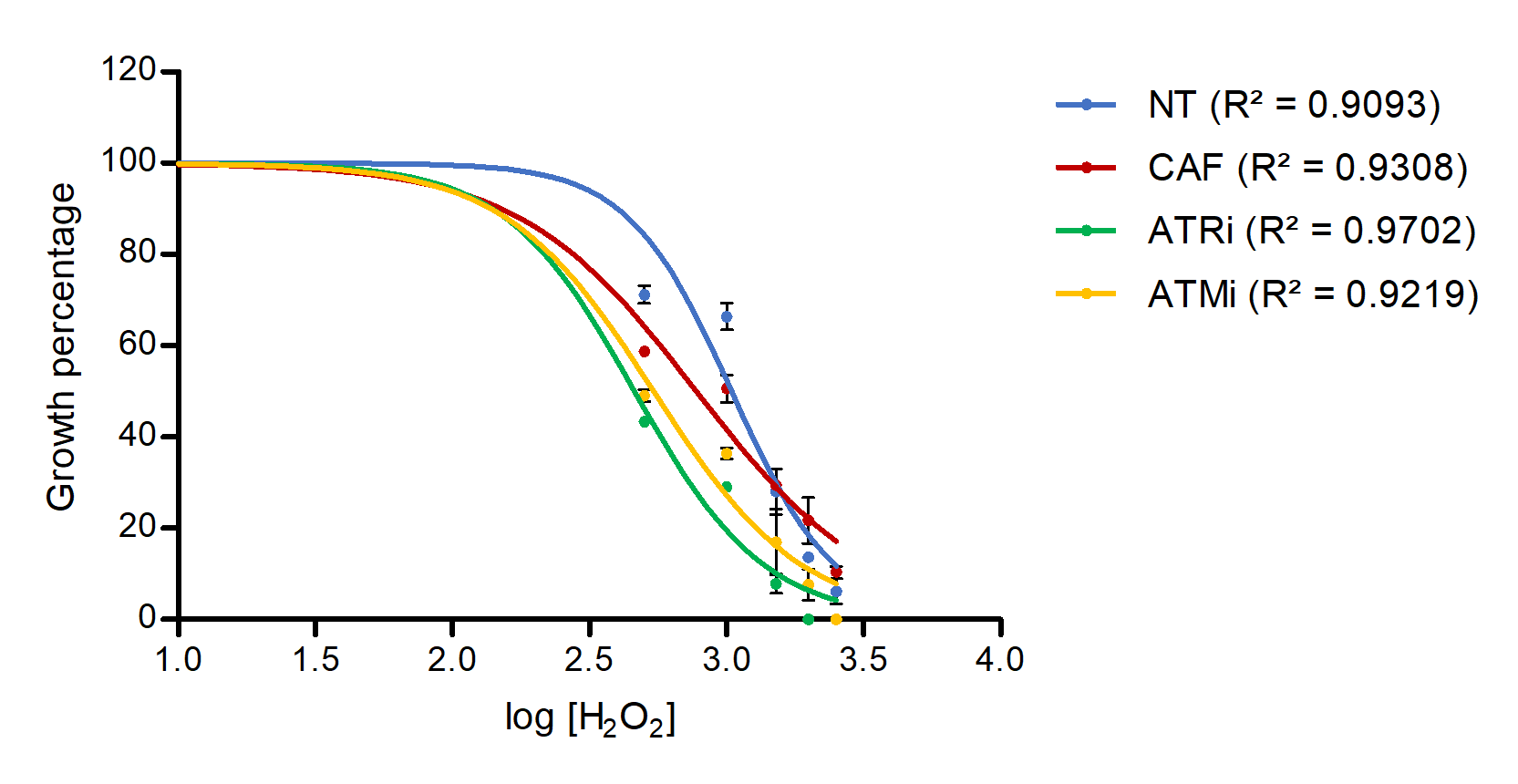

Supplement: S3 Fig — Data from three independent experiments were used to construct the best fit curves for the nonlinear regression calculation of IC50 for H2O2 for cells treated with 10 μM ATRi (VE-821 –green), 10 μM ATMi (KU-55933 –yellow), or 5 mM caffeine (CAF–red), in comparison with cells non-treated by inhibitors (NT–blue). The concentrations of H2O2 used are expressed as log of the concentration in micromolar. The goodness of fit is indicated as R-squared values displayed for each curve. (TIF) [file pone.0205033.s006.tif]
